# Human *Chrysomya bezziana* myiasis: A systematic review

**DOI:** 10.1371/journal.pntd.0007391

**Published:** 2019-10-16

**Authors:** Xianyi Zhou, Dzinkambani Moffat Kambalame, Sitong Zhou, Xiang Guo, Dan Xia, Yemei Yang, Rangke Wu, Juan Luo, Fenglong Jia, Mingchi Yuen, Yuehua Xu, Geyang Dai, Li Li, Tian Xie, Santhosh Puthiyakunnon, Wenxia Wei, Lixian Xie, Siting Liang, Yuqin Feng, Songgen Huang, Yongxuan Hu, Qianzhen Mo, Rongjia Mai, Xiaoqing Zhang, Philip Spradbery, Xiaohong Zhou

**Affiliations:** 1 Department of Dermatology, The Third Affiliated Hospital of Southern Medical University, Guangdong, China; 2 Department of Pathogen Biology, Key Laboratory of Prevention and Control for Emerging Infectious Diseases of Guangdong Higher Institutes, Guangdong Provincial Key Laboratory of Tropical Disease Research, School of Public Health, Southern Medical University, Guangdong, China; 3 Department of Dermatology, Shenzhen Hospital of Southern Medical University, Guangdong, China; 4 The School of Foreign Studies, Southern Medical University, Guangdong, China; 5 Institute of Entomology, Life Sciences School, Sun Yat-sen University, Guangdong, China; 6 Education Technique Center, Southern Medical University, Guangdong, China; 7 Department of Dermatology, Nanfang Hospital, Southern Medical University, Guangdong, China; 8 XCS Consulting, Yarralumla, Canberra, Australia; Hitit University, Faculty of Medicine, TURKEY

## Abstract

**Background:**

Myiasis due to Old World screw-worm fly, *Chrysomya bezziana*, is an important obligate zoonotic disease in the OIE-list of diseases and is found throughout much of Africa, the Indian subcontinent, southeast and east Asia. *C*. *bezziana* myiasis causes not only morbidity and death to animals and humans, but also economic losses in the livestock industries. Because of the aggressive and destructive nature of this disease in hosts, we initiated this study to provide a comprehensive understanding of human myiasis caused by *C*. *bezziana*.

**Methods:**

We searched the databases in English (PubMed, Embase and African Index Medicus) and Chinese (CNKI, Wanfang, and Duxiu), and international government online reports to 6^th^ February, 2019, to identify studies concerning *C*. *bezziana*. Another ten human cases in China and Papua New Guinea that our team had recorded were also included.

**Results:**

We retrieved 1,048 reports from which 202 studies were ultimately eligible for inclusion in the present descriptive analyses. Since the first human case due to *C*. *bezziana* was reported in 1909, we have summarized 291 cases and found that these cases often occurred in patients with poor hygiene, low socio-economic conditions, old age, and underlying diseases including infections, age-related diseases, and noninfectious chronic diseases. But *C*. *bezziana* myiasis appears largely neglected as a serious medical or veterinary condition, with human and animal cases only reported in 16 and 24 countries respectively, despite this fly species being recorded in 44 countries worldwide.

**Conclusion:**

Our findings indicate that cryptic myiasis cases due to the obligate parasite, *C*. *bezziana*, are under-recognized. Through this study on *C*. *bezziana* etiology, clinical features, diagnosis, treatment, epidemiology, prevention and control, we call for more vigilance and awareness of the disease from governments, health authorities, clinicians, veterinary workers, nursing homes, and also the general public.

## Author summary

*Chrysomya bezziana* larvae are characterized by feeding aggressively on the living tissues and body fluids of the host. The dreadful feelings of patients suffering from myiases with severe tissue and bone destruction, even death, and the enormous economic losses in the livestock industries have been described previously. But our findings indicate that *C*. *bezziana* myiases still appear to be under-recognized as a serious medical or veterinary condition throughout the world. Both in China and the world at large, it is probable that *C*. *bezziana* distribution could well be greater than currently reported. Our study provides an opportunity for clinicians and health authorities to gain a comprehensive understanding of this disease from its etiology, clinical features, diagnosis, treatment, epidemiology, prevention and control. In addition, our findings will engage governments, health staff, veterinary workers, aged-care facilities, and also the general public, in efforts to recognize, prevent, and control such infestations.

## Introduction

The Old World screw-worm fly *Chrysomya bezziana*, is an obligate parasite, belonging to the order Diptera, family Calliphoridae, suborder Cyclorrhapha. It is distributed throughout much of southeast Asia, the southern part of east Asia, the Indian subcontinent, Papua New Guinea (PNG), the Middle East, and tropical and subtropical Africa [1, 2]. Myiasis due to *C*. *bezziana* is among the 117 OIE-listed diseases (World Organisation for Animal Health, *http*:*//www*.*oie*.*int/animal-health-in-the-world/oie-listed-diseases-2019/*). The first case of *C*. *bezziana* myiasis was reported in cattle in 1909 [3]. Since then, sporadic cases and even major outbreaks of myiases have been reported globally in animals [4]. Human myiasis caused by *C*. *bezziana* was first reported in 1909 in India [5], and until the present time, at least 291 cases have been reported worldwide in humans (S1 Table). *C*. *bezziana* larvae can cause aggressive and serious destruction of the living tissues, and even bones, of the host, and if vital organs are involved, death may occur [5]. Not only does *C*. *bezziana* cause morbidity and death in animals and humans, but also economic losses in the livestock industries [4, 6]. The intense discomfort experienced by patients with this form of myiasis was described in previous studies [7]. In our study, we will show a comprehensive understanding of human *C*. *bezziana* myiasis, which still remains unclear so far.

## Materials and methods

### Ethics statement

The patient in this manuscript has given written informed consent (as outlined in the PLOS consent form) to publication of their case details.

### Search strategy and selection criteria

We searched the databases relating to *C*. *bezziana* myiasis in humans in English and Chinese up to 6^th^ February, 2019, including PubMed (https://www.ncbi.nlm.nih.gov/pubmed/), Embase (https://www.elsevier.com/solutions/embase-biomedical-research), the African Index Medicus (AIM, http://indexmedicus.afro.who.int/), the China National Knowledge Infrastructure Databases (CNKI, http://www.cnki.net), Duxiu Scholar (http://www.duxiu.com/), and Wanfang (http://g.wanfangdata.com.cn/). We used the following search terms: (chrysomya bezziana) OR (c. AND bezziana) OR (c. bezziana) OR (chrysomya) OR (chrysomya AND bezziana AND villeneuve) OR (chrysomya AND bezziana AND vill) OR (old world screw-worm) OR (old AND world AND screw AND worm) (in English), 蛆症金蝇 or 倍氏金蝇 or 白氏金蝇 or 旧世界螺旋虫(in Chinese). Global government online reports were also reviewed, but the eligible government documents were only available from the Centre for Health Protection, Department of Health, Government of the Hong Kong Special Administrative Region (CHP, http://www.chp.gov.hk/). The websites for Invasive Species Compendia from the Commonwealth Agricultural Bureau International (CABI) (https://www.cabi.org/ISC/) were also searched.

The selection criteria for the literature review were as follows: First, the authors (DMK, XG, STZ, LXX, and XG, YMX, WXW, STL) were assigned to search and select English and Chinese literature, respectively, each step requiring double approval, and if any conflicts arose, they resolved them by consulting the senior authors (XHZ, SP, XYZ, FLJ and MZY). We also consulted some experts in the field when required. Second, after excluding duplicates, all literature was then screened by title, abstract and full-text (S1 Fig), followed by excluding the studies unrelated with *C*. *bezziana*. Third, those reports that were not specified by species identification of *C*. *bezziana* by authors were removed. But the relative records traced from the selected eligible reports’ references or the recommendation of the experts were added. Another ten human cases of *C*. *bezziana* in China (1) and PNG (9) that our team have recorded were also included. All eligible studies concerning etiology, pathology, clinical features and epidemiology of *C*. *bezziana* myiasis, including case reports, case series, reviews, cross-sectional and cohort studies were then retained for the present descriptive analyses (S1 Fig). Datasets of maps were downloaded from the Natural Earth (Free vector and raster map data @ naturalearthdata.com.). Maps of the geographical distribution of *C*. *bezziana* were compiled using Adobe Illustrator CC 2017.

## Results and discussion

### Study eligibility results

Our search of PubMed, Embase, and African Index Medicus databases in English identified 898 possible records of *C*. *bezziana* myiasis. After culling duplicates and checking species identification of *C*. *bezziana*, 99 records of human myiases were confirmed. Among them, 4 human cases reported in Algeria [8], Turkey [9], Spain [10], and Mexico [11] were excluded on the advice of taxonomic experts due incorrect identifications. CNKI, Duxiu Scholar, and Wanfang search in Chinese identified a further 113 records of which 26 were due to *C*. *bezziana*. Myiasis-associated information was also obtained from the periodical known as *Communicable Diseases Watch* (CDW) in which 37 records documented by the government of Hong Kong Special Administrative Region, China are included. The advice of experts added a further 44 records with a grand total of 202 cases. The PRISMA flowchart and checklist are given in S1 Fig.

### Biology/life cycle

**Fig 1:**
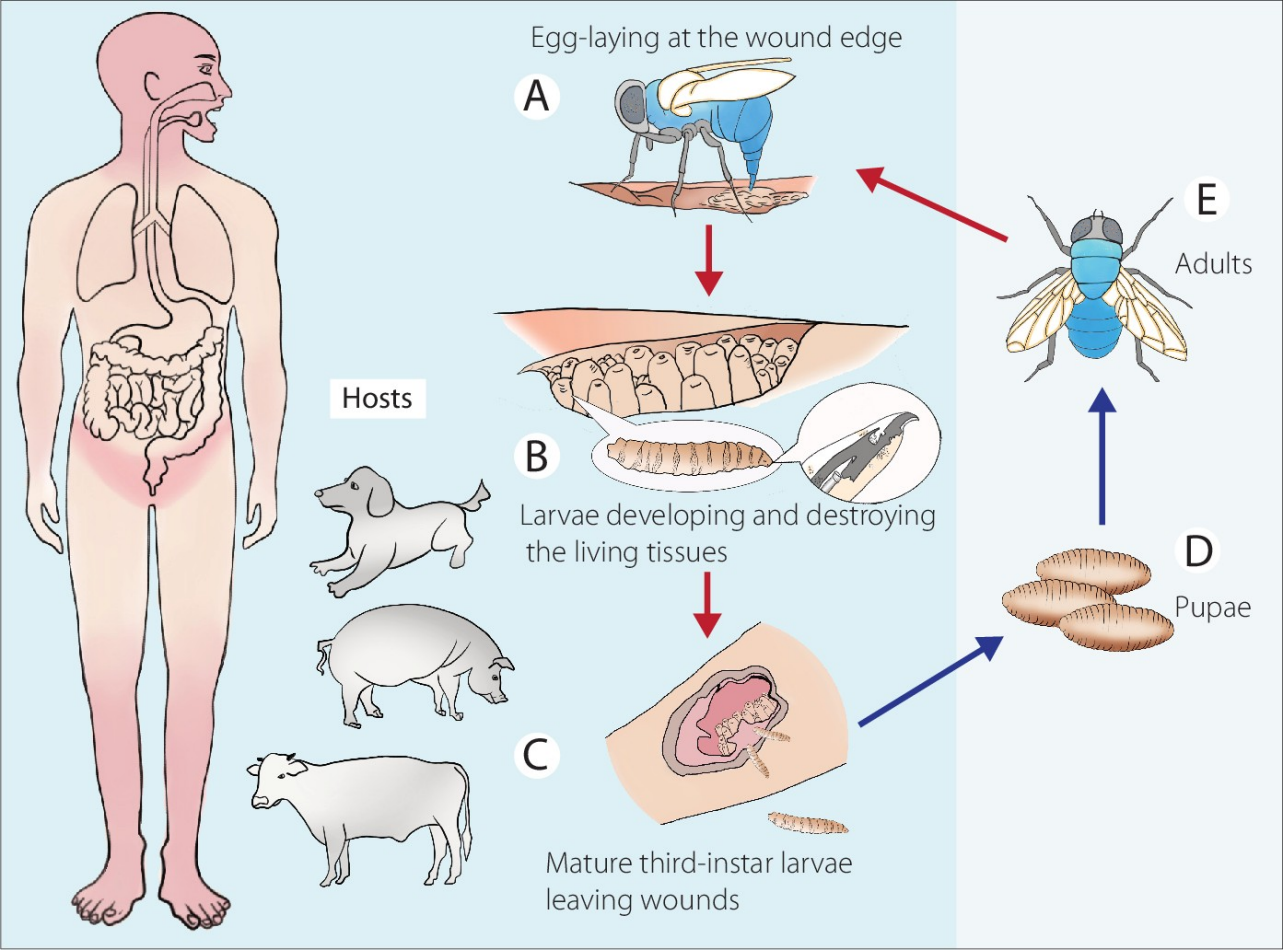
Life cycle of *Chrysomya bezziana*. Referred from Spradbery (2002) [12]. Zoonotic myiasis caused by *C*. *bezziana* involving hosts including warm-blooded animals and human beings. (A) After mating, the gravid females lay egg batches on the edge of wounds, the main infestation sites of humans represented in pink. (B) After hatching, the larvae undergo three stages of development as they feed on blood and wound exudates, and aggressively destroy the living tissues. (C) Mature third-instar larvae evacuate wounds and burrow into soil where they pupate (D) and later emerge as adults (E).

The principle hosts of *C*. *bezziana* are large domesticated animals and native wildlife, and occasionally humans [1, 4, 12]. Although its life cycle has been described in detail by Spradbery (2002) [12], we emphasize the importance of this zoonotic myiasis posing a risk to human beings as well as other warm-blooded animals in Fig 1. *C*. *bezziana* females are attracted to hosts with wounds or moist body openings, including the navel of newborn animals, where batches of up to 245 eggs are laid (Fig 1A). In the case of humans, the infestation sites are mainly the mouth, limbs, perineal and inguinal regions, ear, eye, nose, face, scalp, and torso (S1 Table). Eggs hatch within a few hours and the resulting larvae burrow into the flesh and destroy the living tissues (Fig 1B). After moulting through three larval instars, the mature larvae evacuate the wound after 6–7 days (Fig 1C), drop to the ground and burrow into the soil where they form a puparium (Fig 1D). Adults emerge subsequently (Fig 1E), depending on ambient temperatures.

### Clinical features

Infestations by *C*. *bezziana* commonly manifest as wound and cavity myiases (S1 Table), consistent with the anatomical classification of human myiasis reviewed by Francesconi and Lupi [13]. If a prospective host has close contact with an infested host, an indirect infestation may occur.

In this study, we divided the reported 291 human cases (S1 Table) into three age groups for further analysis: children aged 14-years and less (Ages ≤ 14), adults aged 15 to 64 (Ages 15–64), and the elderly aged 65-years and above (Ages ≥ 65) (S2 Table).

### Underlying diseases

**Fig 2:**
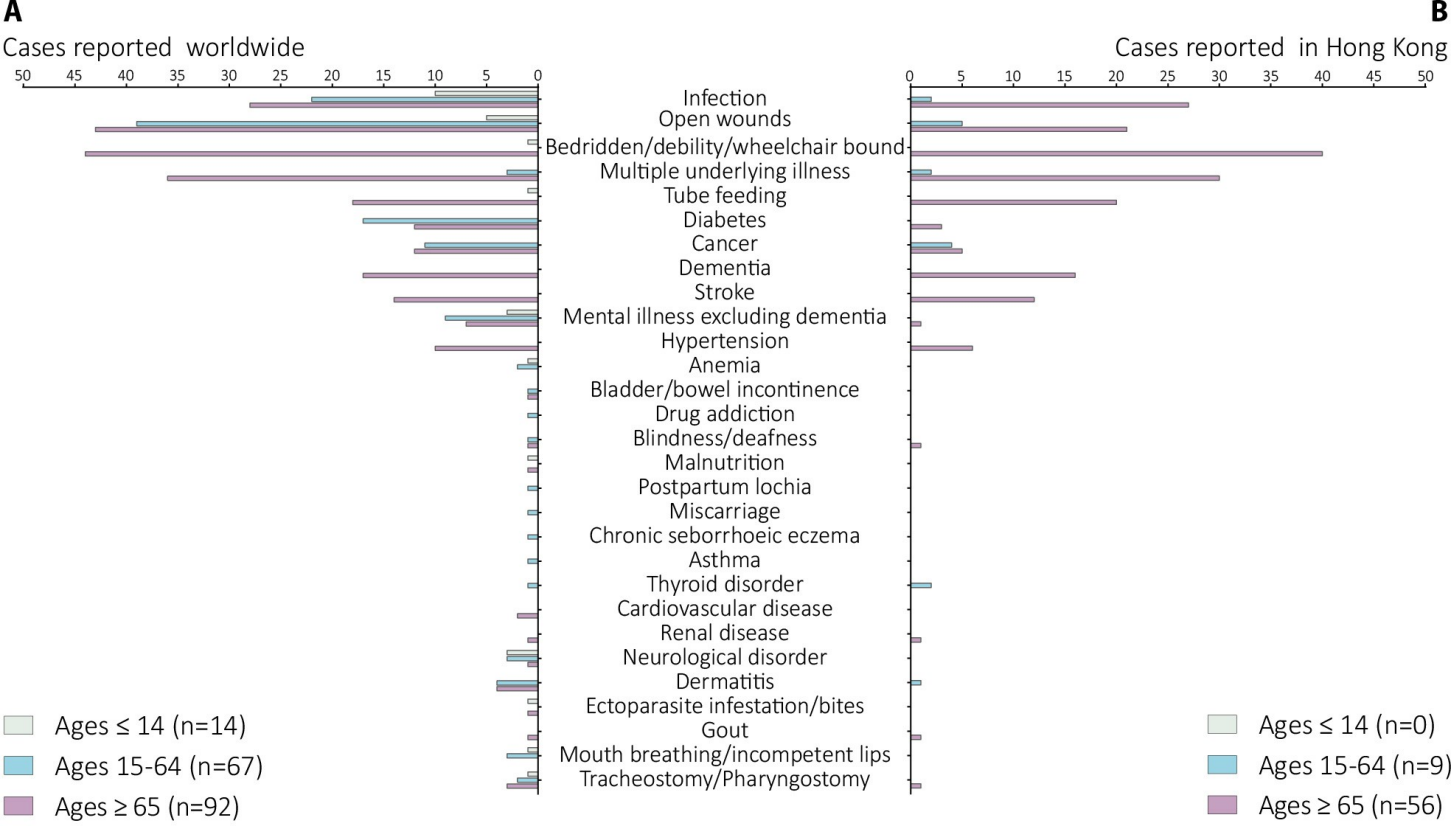
Underlying diseases and conditions recorded in human cases due to *Chrysomya bezziana*. (A) Worldwide. (B) Hong Kong. Data were retrieved from 173 and 65 human cases reported in the world and Hong Kong, respectively. Infection: caused by different types of pathogens such as parasites, bacteria, viruses, and fungus; Mental illnesses excluding dementia: described as mental retardation, schizophrenia and related diseases; Neurological disorders: including epilepsy, seizure, neuro-degenerative disorder, cerebral palsy, quadriplegia, and kyphoscoliosis; Ectoparasite infestations/bites: described as pediculosis and leech bites; Open wounds: including ulcers, wound, trauma, burns, bed sores, lesions, and orbit postevisceration; Cancer: recorded as cancer, carcinoma, tumor, leukemia, and lymphoma.

A total of 173 human cases due to *C*. *bezziana* worldwide have been recorded with underlying diseases and conditions (Fig 2A and S1 Table). Among them, open wounds and infections were most commonly recorded. Eighty-seven patients, including 43 of 92 in Ages ≥ 65, 39 of 67 in Ages 15–64, and 5 of 14 in Ages ≤ 14, had open wounds. Meanwhile, 60 patients, including 28 of 92 in Ages ≥ 65, 22 of 67 in Ages 15–64, and 10 of 14 in Ages ≤ 14, presented with different infections, including serious tropical infectious diseases such as filarial lymphedema, malaria, ankylostomiasis, leprosy, and tuberculosis, human immunodeficiency virus (HIV) infection, sepsis, gangrene, pneumonia, chest infection, bronchitis, pleuropneumonia, endophthalmitis, otitis, rhinitis, sinusitis, pansinusitis, herpes simplex stomatitis, dental abscess, chronic pericoronitis, cellulitis, appendicitis, vulvitis and vaginitis, hemolytic streptococcal infective endocarditis, herpes zoster ophthalmicus, hepatitis B virus infection, perianal condylomata acuminata, aspergillosis, and chromoblastomycosis.

Additionally, elderly patients in Ages ≥ 65 exhibited age-related conditions with 44 of 92 being bedridden, wheelchair bound, or debilitated, 36 suffering from multiple underlying illnesses, and 18 being tube fed. The age-related diseases (ARDs) and noninfectious chronic diseases (NCDs) such as dementia (17) and other mental disorders (7), stroke (14), cancer (12), diabetes mellitus (12), and hypertension (10) were commonly recorded in Ages ≥ 65. The latter NCDs, were commonly reported in Ages 15–64 as well, including diabetes mellitus (17), cancer (11), mental illness excluding dementia (9) and neurological disorders (3). Multiple underlying illnesses (3) and tracheostomy (3) were also recorded. In addition, miscarriage (1) [14], HIV and hepatitis virus infections complicated with mediolateral episiotomy (1) [15], and postpartum lochia (1) [14], during pregnancy, childbirth, and the puerperium were documented among women of childbearing age. Otherwise, drug addiction (1) [16] and chronic seborrhoeic eczema(1) [17] in a German tourist travelling to a Malaysian island in 2012, were also recorded. These data indicated a high risk of *C*. *bezziana* infestation confronting vulnerable individuals. In Ages ≤ 14, the most commonly reported conditions were infections (10), especially ear infections (4 of 11), and additionally those children presenting with mental illness (3) and neurological disorders (3), mouth-breathing or incompetent lips (1), debility (1), tube feeding (1), and pharyngostomy (1).

### Infestation sites

**Fig 3:**
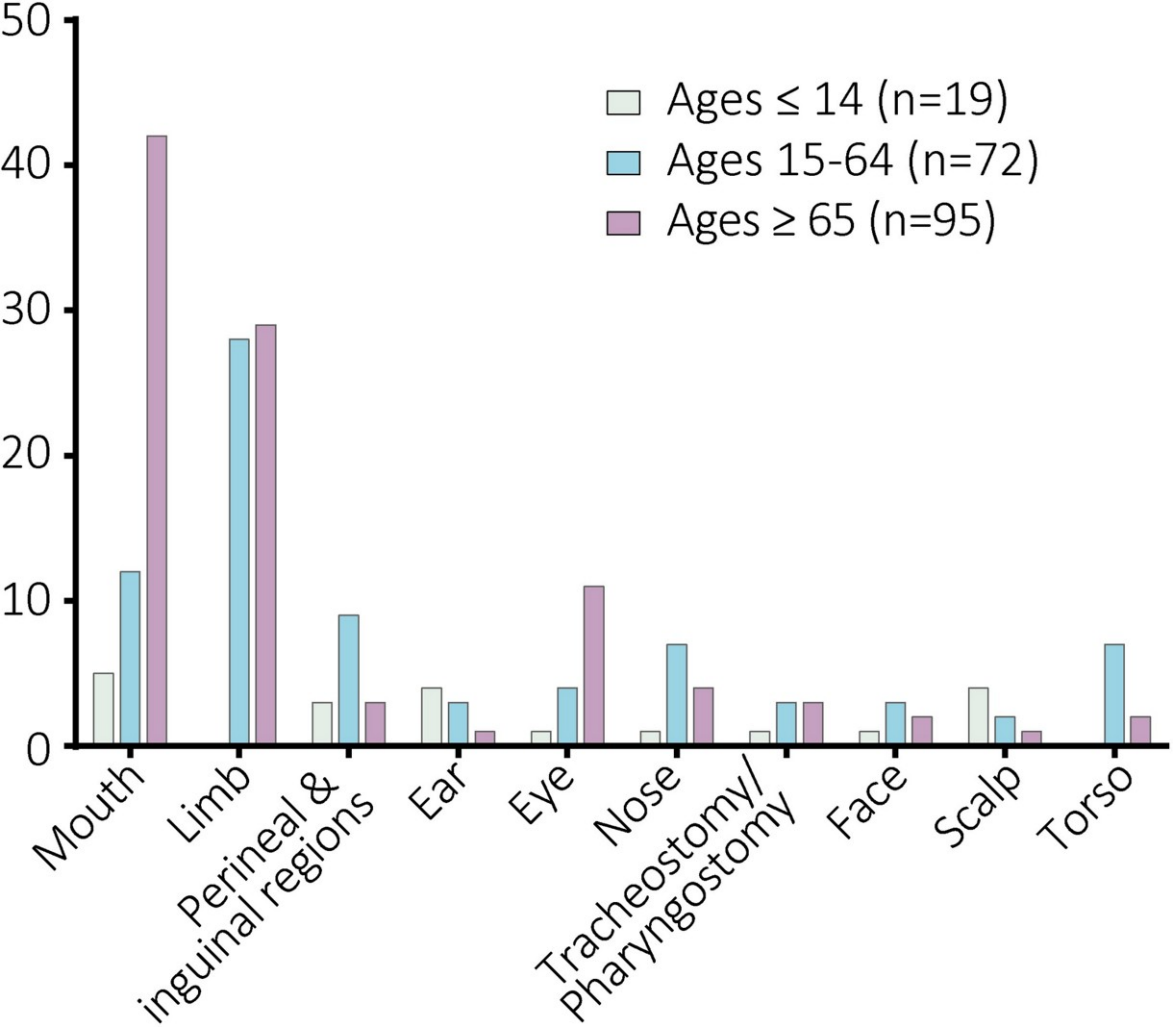
Site of infestations recorded in human cases due to *Chrysomya bezziana*. Data were summarized from 199 human cases worldwide.

Data regarding the site of infestation were summarized from 199 human cases worldwide (Fig 3 and S1 Table). Among them, 60 and 59 cases of myiasis most commonly occurred respectively in the mouth and limbs, especially the lower limbs. In Ages ≥ 65, the most common infestation sites were mouth (42 of 95), limbs (29 of 95), and eye (11 of 95). In Ages 15–64, limbs (28 of 72) were the most common site, followed by mouth (12 of 72), perineal and inguinal regions (9 of 72), nose (7 of 72), and torso (7 of 72) including breast (3), back (1), shoulder (1), buttock (1), and umbilical region (1). While in Ages ≤ 14, the mouth (5 of 19), ear (4 of 19), scalp (4 of 19), and perineal and inguinal regions (3 of 19) were commonly involved.

More severe complications may occur when myiases develop in the head and neck regions. In this study, nine cases recorded the complete destruction of eyes by aggressive *C*. *bezziana* larvae in Hong Kong, India, Indonesia, and Iran, five of them suffering from eye cancer [5, 18–24], while Sachdev et al [19] reported rapid destruction of the eye within two days in a healthy and non-compromised patient. The destruction of tissues and multiple organs involving the eyes, nose, ear, and mouth resulted in death [5]. Likewise a loss of function, or even amputation might occur [25–28]. In addition, seven cases of myiasis occurred in the wounds around tracheostomy or pharyngostomy tubes in a 3-year-old girl and six elderly patients (Fig 2 and S1 Table) [29–34], which put the patients at risk of a probable airway obstruction or aspiration pneumonitis by inhaling the screw-worms, or even aggressive larval invasion of the major blood vessels in the neck.

Further analyses indicated that some underlying diseases and conditions were more commonly involved with specific infestation sites, and vice versa (S2 Fig). Although infections were observed in all types of infestation sites, the sites with higher occurrence rate of infections involved the main facial organs including ear (7 of 8), nose (8 of 10), and eye (10 of 15), and perineal and inguinal regions (5 of 11). Meanwhile, the total of 15 eye infestations were commonly associated with open wounds (7), cancer (6), multiple underlying illness (5), and bedridden, wheelchair-bound, or debilitated patients (5). Four out of five facial infestations suffered from cancer. Fifty-five mouth infestations were most commonly associated with mouth breathing or incompetent lips (4 of 4), tube feeding (18 of 19), bedridden, wheelchair-bound or debilitated (33 of 45), neurological disorder (5 of 7), dementia (11 of 17), multiple underlying illnesses (25 of 39), stroke (7 of 14), other mental disorders (8 of 19), infections (16 of 60), and cancer (7 of 23). Fifty-seven limb infestations were recorded with dermatitis (7 of 8), diabetes mellitus (22 of 29), open wounds (48 of 87), and infections (12 of 60). Among them, 22 of 29 patients with diabetes mellitus, the infestation most commonly occurred in the lower limbs due to their diabetic feet (S1 Table) [25–27, 35–37]. Five of six patients with filarial lymphedema presented with infestations in their lymphedematous limbs (S1 Table) [37, 38]. Among 23 underlying cancer patients, the infestation sites commonly occurred in the face (4 of 5), following tracheostomy or pharyngostomy (4 of 7), eye (6 of 15), mouth (7 of 55), and torso (3 of 9).

### Clinical symptoms

The gross pathological changes due to myiasis relate to the developmental stages of the larvae as they feed on the host. The larvae destroy living tissues and cause deep, painful, ulcerative lesions associated with bleeding and a serosanguinous purulent discharge. Secondary infections, fever, weight loss, and inflammation may consequently occur. A massive initial infestation or a series of repeated strikes can lead to enormous soft tissue destruction and wound extension [39]. The larvae can destroy bones, nasal sinuses, orbital cavities, hard palate, eyeballs, hearing apparatus, and teeth (S1 Table). Such aggressive invasion of the host body can lead to serious complications including debility, limb amputation [25–28], blindness [5, 18–24], and death [5].

Apart from an ulcer or a wound filled with living larvae (120 of 144), the symptoms of *C*. *bezziana* myiasis were mostly non-specific, ranging from pruritis and pain, to severe tissue and/or bone destruction (S3 Fig and S1 Table). The other commonly reported symptoms included bleeding (49), ulcer, wound, tunnels or perforations (49), discharge (45), swelling (38), pain (37), fever (30), necrosis (27), severe tissue and/or bone destruction (23), and a foul smell emanating from the wound (21). The skin surrounding the infested wound could present with inflammation, swelling, redness, and cellulitis. The patients with oral cavity myiases commonly had a foul smell, including halitosis. The ulcers could get large rapidly and these extensive ulcers may be associated with serious complications [25–28]. For instance, an 89-year-old lady from Hong Kong suffered from below-knee amputation due to the extensive damage [25].

### Diagnosis

**Fig 4:**
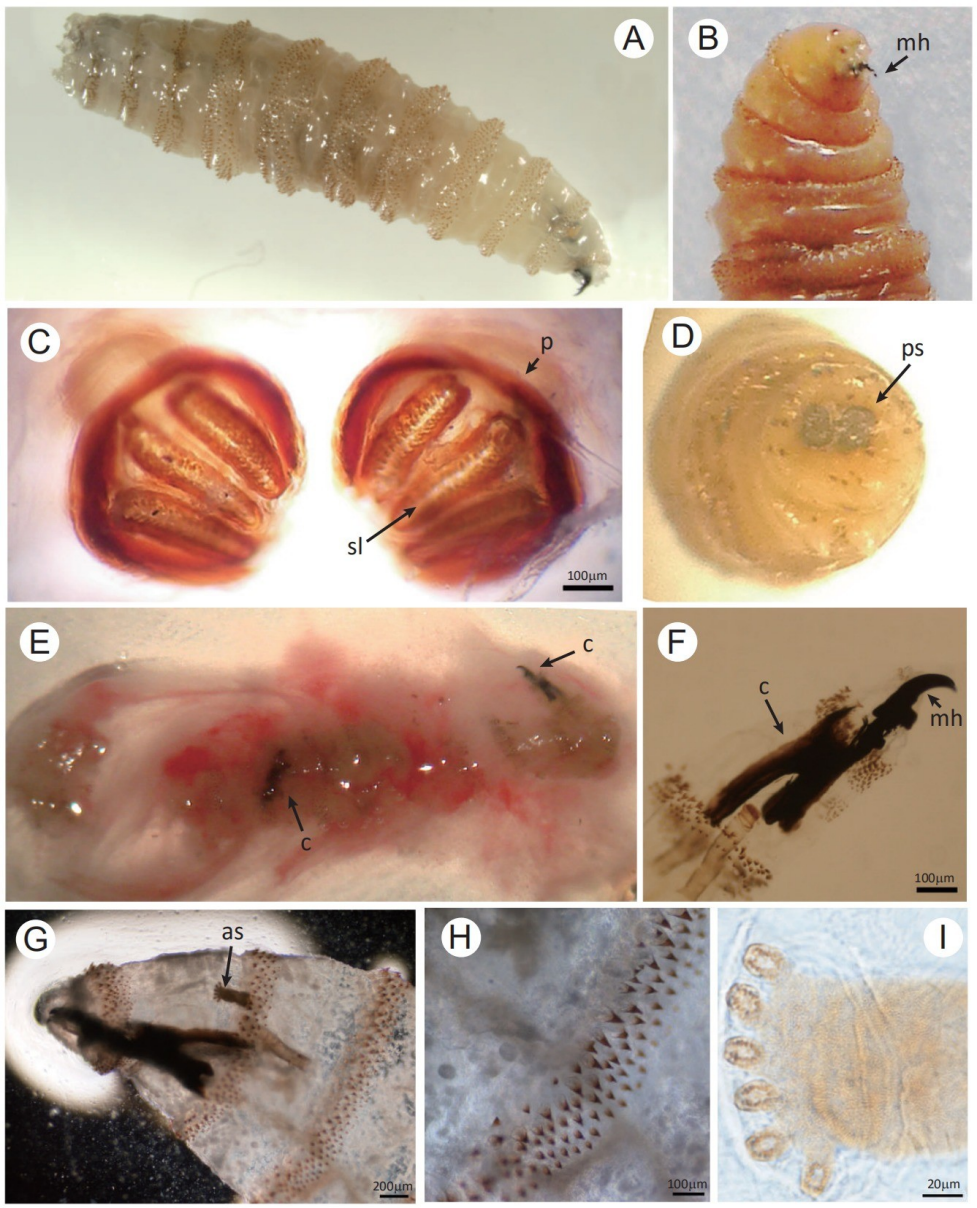
Morphology of the third-instar larva of *Chrysomya bezziana*. (A, B) The third-instar is approximately 14 mm in length with strong mouth hooks (mh). (C) The posterior spiracles (ps) are observed with the spiracular slits (sl) slightly convergent and the peritreme (p) thick and incomplete. (D) Dissecting micrograph of the posterior spiracles (ps). (E-I) Two third-instar larvae were isolated from the discharge extruded from the patient’s skin lesion. (E) Showing the discharge containing two larvae with two black cephalopharyngeal skeletons (c), (F and G) the strong and robust mouth hook (mh), (G and I) the anterior spiracle (as) with palmate shape due to six papillae arranged in single row, and (H) the intersegmental spines with the single, darkened and tapered tips recurved toward the body. Bar: C, F and H = 100 μm; G = 200 μm; I = 20 μm.

The gold standard for diagnosis of *C*. *bezziana* myiasis is entomological evidence for species identification. The sampled larvae are killed by immersion in near boiling water (90–100°C) for 30secs before being preserved in 70%-95% ethanol (reviewed by Francesconi and Lupi) [13]. The anatomical features of *C*. *bezziana* larvae can be used for its initial identification: the body shape, body surface with prominent bands of thorn-like spines, papillae, spiracles (posterior and anterior), dorsal tracheal trunks, mouth hooks (mh), and cephalopharyngeal skeleton (Fig 4) [40, 41]. See the diagnostic manual by Spradbery [1, 12] and papers by Sukontason et al [41] and Gan [42] for further details.

Alternatively, the larvae can be reared to adults followed by morphological identification using the adult taxonomic keys [12, 43]. The real-time PCR is widely used for adult fly surveillance and identification of *C*. *bezziana* [44, 45]. Computer tomography (CT) or magnetic resonance imaging (MRI) have been mainly utilized to locate the larvae and delineate the extent of erosion, especially when the disease involves sensitive body parts including the mouth, nasopharynx, and eye[21, 46–48].

### Treatment

The most important and effective treatment is the removal of all living larvae of *C*. *bezziana* quickly and thoroughly from the infested sites [13]. Early and proper treatment is essential to help wound healing and stop the rapid and destructive process of *C*. *bezziana* myiasis. Alhady et al reported that a 9-year-old boy presenting with severe ophthalmomyiasis due to *C*. *bezziana* which resulted from his primary aural myiasis, stressing the need for early and thorough larvae removal [49]. Treatment options depend on the different sites of infestation and different degrees of damage (S1 and S3 Tables).

First, manually remove all visible larvae using tweezers or forceps followed by debridement. This method can be used to treat myiases affecting the majority of infested body sites (S3 Table).

Second, the use of suffocating agents including turpentine oil, mineral oil, vaseline, liquid paraffin, petroleum jelly, and bee wax (reviewed by Nene et al [50]) are convenient and effective for patients, especially at the primary care level. These agents force the larvae out by blocking air entry [50]. This method proved to be effective where the larvae were successfully removed from most infested sites, even complex deep structures, including limb (13), mouth (11), eye (6), nose (4), perineal and inguinal regions (4), face (3), ear (2), scalp (2), and even the tracheostomy or pharyngostomy wounds (S1 and S3 Tables). The turpentine oil was commonly used as the effective suffocating agent [15, 33, 50–55]. For instance, cotton buds or gauze impregnated with turpentine oil were usefully applied in the myiases treatment involving limbs, mouth, eye, ear, nose, facial tumor base [50, 51, 53–55], and perineal and inguinal regions [15, 52]. But if the occlusive measure was applied to infested sites around tracheostomy and pharyngostomy tubes, this increases the risk of chemical pneumonitis [33].

Third, surgical removal that involves extensive wound exploration under anesthesia depending on the degree of tissue damage. Infestations in the mouth (18), limbs (12), eyes (10), ears (3), and tracheostomy/pharyngostomy wounds (3) were treated by surgery. Fourteen of them were recorded with serious necrosis, severe tissue and/or bone destruction, nine patients received the eye exenteration [5, 19–24], and four underwent limb amputation [25–28] (S1 Table).

Moreover, a single dose of 200 μg/kg of ivermectin was suggested as a potential adjunctive treatment after larval removal for severe *C*. *bezziana* myiasis [56, 57]. Meanwhile, ivermectin was reported to treat severe orbital myiases due to *Cochliomyia hominivorax* prior to surgery, thereby preventing enucleation or further damages to deeper tissues [58]. However, due care should be taken in giving the anti-parasitic therapies as these could result in larvae dying in situ within the host body. Vigilance must be kept to avoid secondary complications due to decomposition of larvae. Due to no double-blind clinical trial to evaluate the efficacy of ivermectin use on myiases [13], oral ivermectin therapy still needs to be appropriately selected depending on the severity and location of the infested sites of myiasis. In addition, antibiotic therapy, nutritional support and maintenance therapies against any underlying diseases should be prioritized for those patients in need.

### Outcomes

In this study, 171 patients had documented outcomes which varied with their health status and infestation sites (S1 and S4 Tables). The majority (148) of them had positive outcomes after effective treatment, but 23 died (S4 Table). Even among the majority (148), four patients were subject to limb amputations [15–18] and seven eye exenterations [20–25]. Among the mortality cases (23), 22 occurred in Ages ≥ 65, and only one in Ages 15–64. Twenty out of 23 were recorded with multiple underlying diseases, including a patient in Ages 15–64, who was a 39-year-old Indian woman suffering from malaria, ankylostomiasis, etc., and her death was attributed to the worsened multiple underlying diseases and extreme exhaustion together with rapid and heavy destruction of the main facial organs caused by thousands of screw-worms (S1 Table) [5]. Infested sites in the patients that died were the mouth (20 of 23), eyes (2 of 23), nose (2 of 23), face (1), and torso (bed sores) (1) (S4 Table). The cause of death was recorded including pneumonia (10 of 23), myocardial infarction (3 of 23), and sepsis (3 of 23). Therefore, clinicians should bear in mind that prompt and proper management of both myiasis and underlying diseases, especially for the elderly, is the key to improved outcomes.

### A case report of human *C*. *bezziana* myiasis in the elderly individual

**Fig 5:**
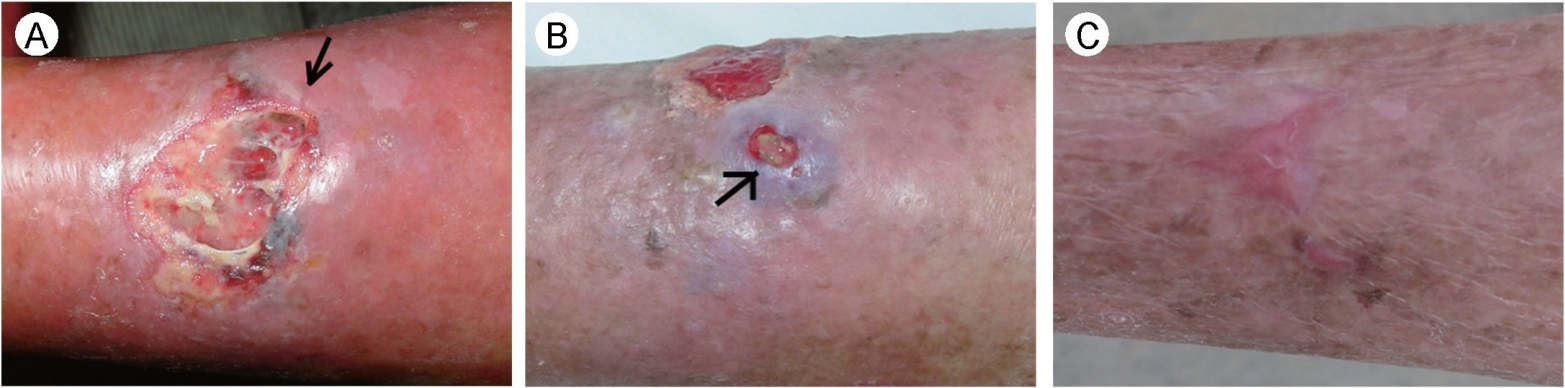
Photographs showing the healing process of the skin ulcer of the patient in Guangdong. (A) The skin ulcer on the patient’s left leg showing a large ulcer measuring 4 cm × 5 cm with undermining up to 8 cm × 8 cm; the black arrow shows tunnels with the third-instar migrating in. An obvious flare expanded to her whole left limb. (B) The skin ulcer showing a grayish-blue skin nodule that appeared near the ulcer, ф1.5 cm, the black arrow shows two larvae in the discharge. (C) The ulcer healed completely.

An 84-year-old female from Lufeng County, Guangdong Province, China was diagnosed with wound myiasis due to *C*. *bezziana* at our laboratory (Guangzhou). The patient was referred to Nanfang Hospital in Guangzhou due to the lack of experience in the rural clinic. She had a history of hypertension for more than 20 years and cerebral thrombosis for 3 years. Her main complaints were the sensation of larval movement, skin ulcer, unbearable and intense pain, and she was extremely agitated and fearful and persistently sleepless. Four days prior to her referral, she sustained a scratch with bleeding on her left leg, but her wound quickly progressed to form a large and deep undermining ulcer reaching the muscle layer (Fig 5A). The patient presented with an increased white blood cell count of 14.14×10^9^/L, a neutrophilic granulocyte (NEU) count of 10.87×10^9^/L, a monocyte count of 0.69×10^9^/L, an eosinophil (EOS) count of 0.61×10^9^/L, a NEU percentage (NEU%) of 76.8%, a C-reactive protein level of 10.7 mg/L and an IgG level of 35.5 g/L. Both *Enterobacter cloacae* and *Stenotrophomonas maltophilia* were isolated from the purulent discharge.

We diagnosed the wound as a *C*. *bezziana* myiasis by species identification using the living larvae collected from the ulcer (Fig 4B–4D), and also the dead larvae extracted from the lesion mixed with puss, blood and mucous discharge (Figs 4E–4I and 5B). The dead larvae were fixed in 70% ethanol, followed by submerging in 10% KOH for 24 hours. This procedure resulted in the effective isolation of the dead larval exoskeletons from the discharge, which assisted species diagnosis (Fig 4E–4I).

Treatment included removal of larvae by manual and vaseline ointment occlusion methods, and wound debridement, with antibiotic and anti-hypertensive treatment as well as nutritional support. The vaseline ointment was promptly smeared over the ulcer and applied at more than five mm thick for 24 hours. After only half an hour, the patient’s pain was alleviated and her condition improved significantly. Eight third-instar larvae crawled out of the ulcers in 10 hours. Soon her ulcer began to heal (Fig 5B). Although the patient was managed promptly with experienced doctors in our hospital, the myiasis ulcers with secondary bacterial infection were not completely healed until three weeks after antibiotic treatment, and complete wound healing took about three months (Fig 5C). In this case of a frail 84-year-old patient, her invasive injuries could have been reduced more quickly and her suffering would have been curtailed, if prompt diagnosis and proper treatment had been provided at the primary health care.

### Epidemiology

#### Species distribution

**Fig 6:**
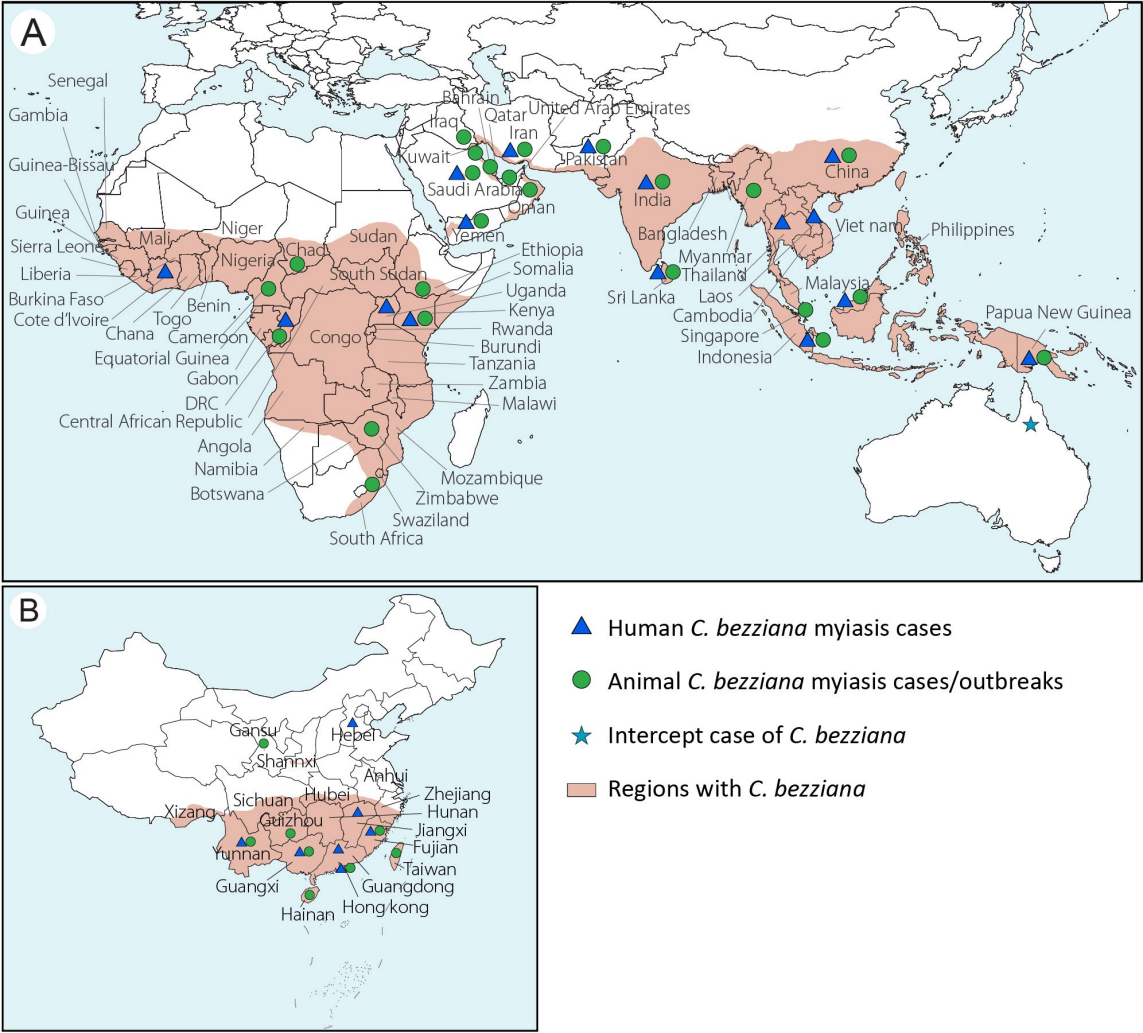
Geographical distribution of *Chrysomya bezziana* and myiasis caused by *C*. *bezziana*. (A) Worldwide; (B) China. The maps were constructed using Adobe Illustrator.

Global distribution of *C*. *bezziana* has been described by Animal Health Australia (AHA, 2019) [1] and CABI [4], which plotted for 63 countries (Fig 6A). In the present study, the occurrence of *C*. *bezziana* has been summarized for at least 44 countries worldwide in the published literature (S5 Table and Fig 6A). Meanwhile, *C*. *bezziana* has been intercepted by quarantine in Australia [59]. However, there is a possibility of permanent colonization of new geographical areas by this species after accidental introduction, either through an infested host via ship or aircraft, and also returning livestock vessels [60]. For instance, some countries in the Middle East, such as Bahrain, Kuwait, Iraq, and Yemen have been colonized by *C*. *bezziana* after accidental introductions [4]. In addition, the spread of human myiasis cases may be associated with aircraft travel [4].

In China, *C*. *bezziana* was found in 17 provinces/province-level regions including Hainan, Fujian, Guangdong, Guangxi, Yunnan, Guizhou, Sichuan, Qinghai, Xizang, Hunan, Hubei, Jiangxi, Gansu, Shannxi, Hebei, as well as Hong Kong and Taiwan (Fig 6B and S5 Table).

#### Human myiasis reports worldwide

In the present study, 190 patients had records of age (S2 Table), 193 of gender (S6 Table), and 165 with their socioeconomic status (S7 Table). Worldwide, *C*. *bezziana* myiasis commonly affects old people with 96 recorded in Ages ≥ 65, 73 in Ages 15–64, and 21 in Ages ≤ 14 (S2 Table), while 154 patients (154 of 165, 93.3%) were of low socioeconomic status or living in aged-care homes, etc (S7 Table). But no obvious differences were found in the gender analysis (S6 Table).

Between 1909 and 2019, 16 countries worldwide have recorded 291 human cases of *C*. *bezziana* myiasis (Tables 1 and S1 and Fig 6A). According to the published literature, Asia has reported by far the highest number of cases 94.5% (275 of 291), with most distributed in China 36% (99 of 275) (85 cases recorded in Hong Kong, 14 in mainland China) (Fig 6B), and India 36% (99 of 275), followed by Sri Lanka 15.6% (43 of 275), and Iran 4.7% (13 of 275). Of these, 111 of 275 were reported in current decade in Asia. Meanwhile, Oceania and Africa only reported 3.1% (9 of 291) and 2.4% (7 of 291), respectively, and all seven human cases in Africa and six in Oceania were recorded before 1968.

**Table 1:** Number of human cases reported in each continent in different time periods.

Continent	Country/Region	-1968	1969–1978	1979–1988	1989–1998	1999–2008	2009–2019	Total
East Asia	China	3	4	4	0	42	46	99
South Asia	India	60	1	0	7	7	24	99
	Sri Lanka	2	0	0	14	0	27	43
	Pakistan	0	0	0	0	0	1	1
Middle East	Iran	0	1	0	1	3	8	13
	Yemen	0	0	0	0	8	0	8
	Saudi Arabia	0	0	2	0	0	0	2
Southeast Asia	Malaysia	0	0	1	0	3	2	6
	Laos	0	0	0	0	0	1	1
	Thailand	0	0	0	0	0	1	1
	Indonesia	0	0	0	0	1	1	2
Africa	Uganda	3	0	0	0	0	0	3
	DRC	2	0	0	0	0	0	2
	Kenya	1	0	0	0	0	0	1
	Cote d'Ivoire	1	0	0	0	0	0	1
Oceania	PNG	6	0	3	0	0	0	9
	Total	78	6	10	22	64	111	291

PNG = Papua New Guinea, DRC = Democratic Republic of Congo.

#### Human myiasis reports in Hong Kong

In mainland China, there were only 14 human cases reported in six provinces including Fujian [61], Guangdong, Guangxi [14, 62], Yunnan [2], Jiangxi [63], and Hebei [64] (Fig 6B and S1 Table). But 85 human cases have been reported in Hong Kong based on both government documents and the published literature. Since the first human case report in 2002 [48], the government of Hong Kong, through the Center of Health Protection (CHP), has been reporting the monthly occurrence of cases consistently through the periodical CDW. Although *C*. *bezziana* myiasis is not a notifiable disease in Hong Kong, this publication provides a viable opportunity for a better understanding of the epidemiological status of screw-worm in a city. From the data available, the cases appear to be concentrated in Ages ≥ 65, in which 83.8% (56 of 68) of the cases were reported with a mean age of 82.6 years (S2 Table). The most common underlying diseases among the 56 elderly patients in Ages ≥ 65 in Hong Kong were infections, ARDs and NCDs, 30 suffering with multiple underlying illnesses and 40 bedridden or wheel-chair bound, 27 with infections, 16 with dementia, 12 with stroke, 20 with feeding tubes, while 21 patients had open wounds (Fig 2B and S1 Table). Hong Kong is a developed city and has an aging society. In 1996 and 2016, the population of aged (the percentage of Ages ≥ 65) in Hong Kong reached 10.1% and 16%, respectively, and it is expected to increase to 34% by 2066 [65]. Therefore, the urgency for preventing myiasis due to *C*. *bezziana* in the elderly, especially Ages ≥ 65 who are suffering from infections, ARDs or NCDs, being debilitated and living in aged-care homes, cannot be underestimated.

A summary of animal cases and outbreaks caused by *C*. *bezziana* is given in Appendix S1. Thereby, despite the global distribution of *C*. *bezziana* being recorded in 44 countries, human and animal cases were only reported in 16 and 24 countries, respectively. In China, this fly species is distributed in 17 provinces, but only 6 and 8 provinces have reported human and animal cases, respectively. Therefore, both in China and the world at large, it is possible that *C*. *bezziana* distribution could be far greater than currently reported. Sutherst et al [60] predicted that some parts of the Americas and Australia could provide favorable conditions for colonization by *C*. *bezziana* once introduced. The potential for incursions and subsequent spread of *C*. *bezziana* poses a risk to global public health. Furthermore, systematic surveillance and treatment studies are required and these would be expected to provide for the deployment of better *C*. *bezziana* prevention, control, and treatment strategies worldwide.

### Prevention and control

The prevention and control of *C*. *bezziana* myiasis requires an integrated approach that includes personal protection, environmental improvement, good animal husbandry practices, proper keeping of pet animals such as dogs, and legislation including making the condition a notifiable disease.

It is essential to maintain good personal hygiene such as skin and oral care, since blood and wound exudates and their odours can attract gravid females to lay eggs on a host [13]. Especially, all open wounds need to be kept clean and thoroughly dressed, in particular among those patients leaving hospitals with cancerous lesions, feeding tubes, tracheostomy or pharyngostomy, trauma, burns, ulcers, diabetic feet, bed sores, orbit postevisceration, and lymphedematous limbs. We highlight the urgent need for health education for these patients, their families, and health staff, especially in aged-care homes and primary health care centers, in preventing *C*. *bezziana* infestations.

Physical barriers to infestation include using bed nets [13] and stationing screens at all possible points of entry such as air vents, windows and doors [66]. The use of anti-fly curtains, air curtains, and insect electrocutors are recommended [66]. In addition, the appropriate use of insecticides [67] can be used to reduce the risk of *C*. *bezziana* myiasis.

Moreover, respective government agencies can impose quarantine restrictions on *C*. *bezziana* cases from endemic areas. This measure has been adopted by the Australian Government which, through functional surveillance systems and quarantine restrictions, has prevented *C*. *bezziana* incursions into Australia [68]. Governments can also embark on fly eradication programs by using strategies that have proven successful in eradicating the related New World screw-worm fly, the most effective being the sterile insect technique (SIT) [69]. Meanwhile, health personnel should ensure that health care workers, farmers, and the general public are aware of *C*. *bezziana* to enable prompt prevention, diagnosis, and treatment.

### Limitations

Reports from the authoritative government appraisal agency worldwide (apart from the government of Hong Kong Special Administrative Region and Animal Health Australia) and the systematic surveillance were lacking. Therefore human myiasis due to *C*. *bezziana* has the possibility to be underestimated in our study.

### Conclusions

C. *bezziana* myiasis is a devastating and rapidly-progressing condition, posing a risk to public health. Regrettably, this disease appears to have been under-recognized as a serious medical and veterinary condition, for human and animal cases have only been reported in 16 and 24 countries respectively, although it is recorded as present in 44 countries worldwide. Attentions should be raised by the public and relative agencies, especially the aged-home sectors and the primary clinics.

## Supporting information

S1 Fig The PRISMA flowchart. (PDF)

S2 Fig Heat map of the underlying diseases combined with the infestation sites. Cases numbers are depicted as standarized Z-scores, where red represents large number and white represents small number. *Open wounds: including ulcers, wound, trauma, burns, bed sores, lesions, and orbit postevisceration. **Cancer: recorded as cancer, carcinoma, tumor, leukemia, and lymphoma.
(PDF)

S3 Fig Clinical signs and symptoms of human cases due to *Chrysomya bezziana* myiasis. (PDF)

S4 Fig Preferred reporting items for systematic reviews and meta-analysis (PRISMA) checklist. (PDF)

S1 Table Characteristics of human cases due to *Chrysomya bezziana* myiasis recorded worldwide. (PDF)

S2 Table The patients with *Chrysomya bezziana* myiasis were grouped by age. (PDF)

S3 Table Summary of main therapies recorded in human cases with *Chrysomya bezziana* myiasis worldwide. (PDF)

S4 Table Health outcomes of human cases due to *Chrysomya bezziana*. (PDF)

S5 Table Distribution of *Chrysomya bezziana* recorded in the world. (PDF)

S6 Table A gender analysis in patients with *Chrysomya bezziana* myiasis. (PDF)

S7 Table Analysis of the socioeconomic status of patients with *Chrysomya bezziana* myiasis. (PDF)

S1 Appendix Animal cases or outbreaks due to *Chrysomya bezziana* myiasis. (PDF)
