# Supplementary figures and images for: Human Chrysomya bezziana myiasis: A systematic review
Source: PLoS Negl Trop Dis. 2019 Oct 16;13(10):e0007391. doi: 10.1371/journal.pntd.0007391 (PMC6821133; doi:10.1371/journal.pntd.0007391)

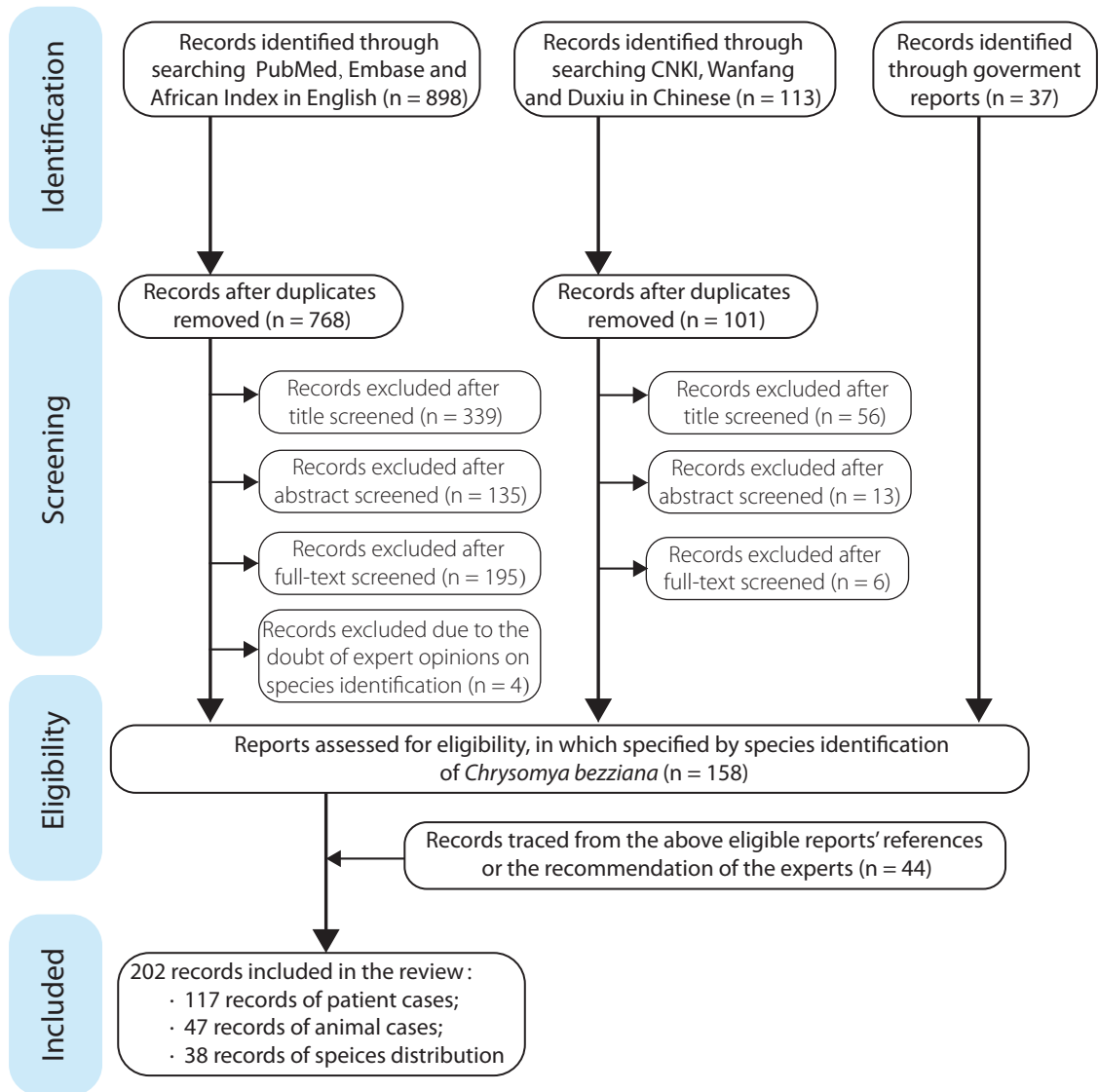

Supplement: S1 Fig — (PDF) [file pntd.0007391.s001.pdf]

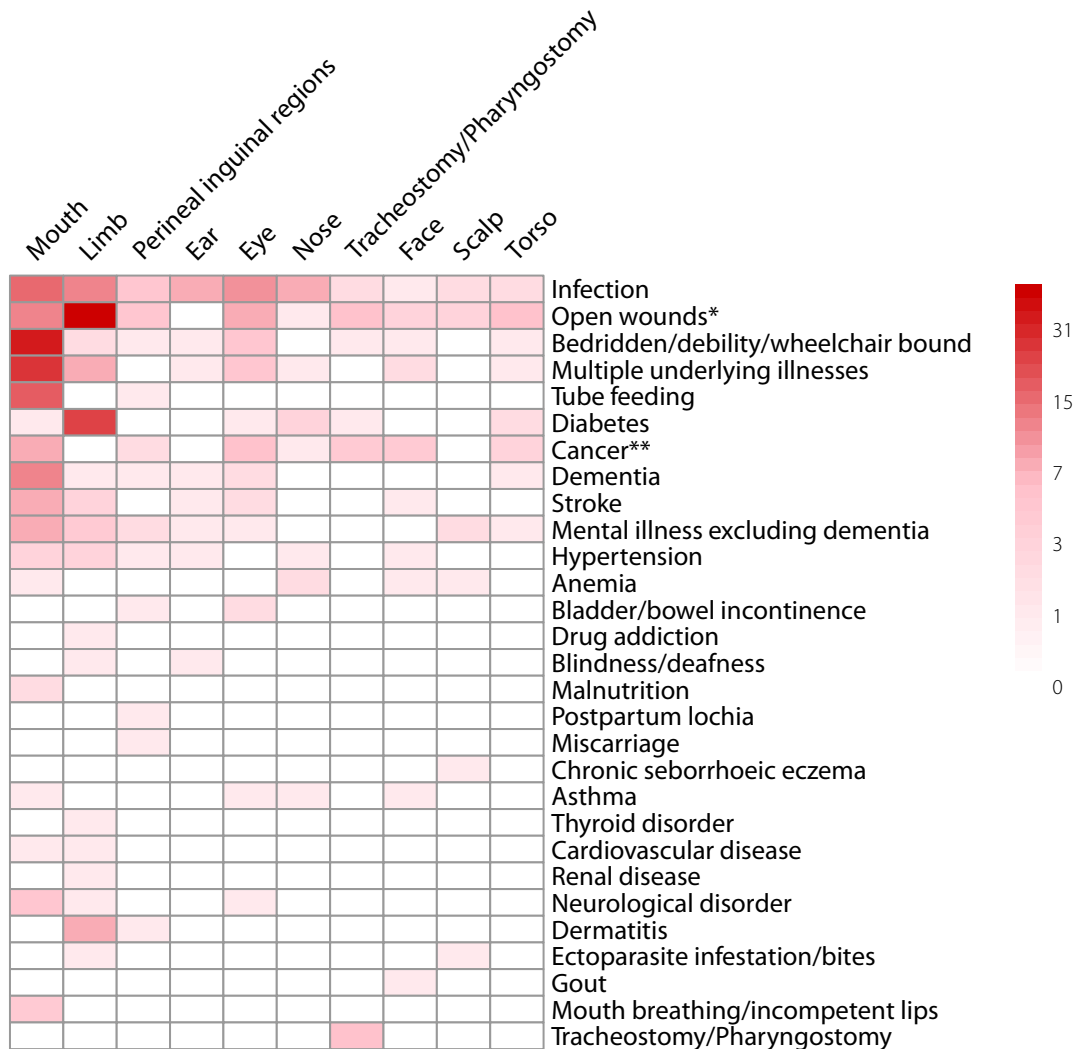

Supplement: S2 Fig — Cases numbers are depicted as standarized Z-scores, where red represents large number and white represents small number. *Open wounds: including ulcers, wound, trauma, burns, bed sores, lesions, and orbit postevisceration. **Cancer: recorded as cancer, carcinoma, tumor, leukemia, and lymphoma. (PDF) [file pntd.0007391.s002.pdf]

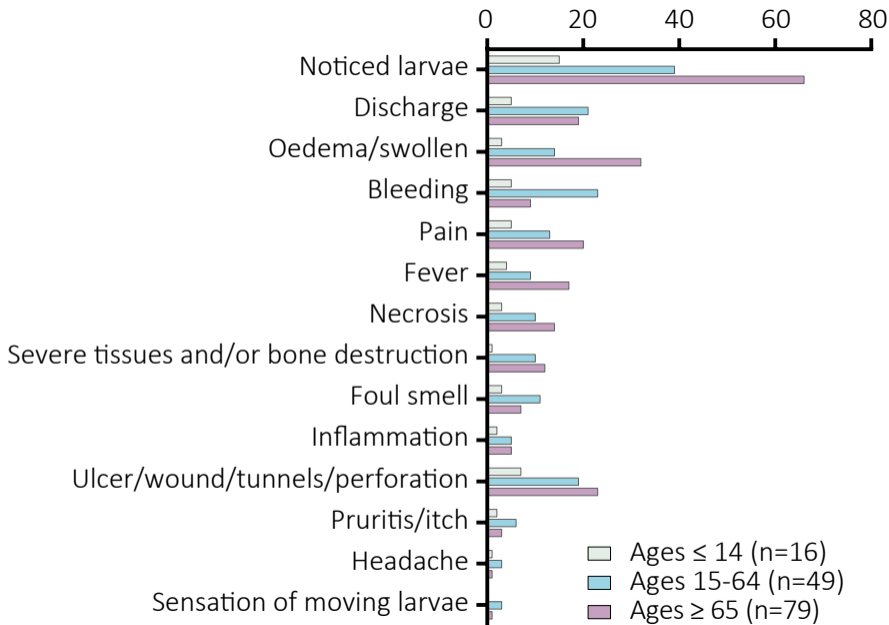

Supplement: S3 Fig — (PDF) [file pntd.0007391.s003.pdf]
