# Supplementary material for: Human Chrysomya bezziana myiasis: A systematic review
Source: PLoS Negl Trop Dis. 2019 Oct 16;13(10):e0007391. doi: 10.1371/journal.pntd.0007391 (PMC6821133; doi:10.1371/journal.pntd.0007391)
Supplement: S1 Table — (PDF) [file pntd.0007391.s005.pdf]

S1 Table. Characteristics of human cases due to *Chrysomya bezziana* myiasis recorded worldwide.

ID	Age	Sex	Year	Country	Location	Socioeconomic status	Site of infestation	Methods for removal of larvae	Underlying diseases	Clinical signs and symptoms	Outcome
1[70]	49	F	2004	China	Hong Kong	The elderly home	Torso: left breast	Surgical removal	Left breast cancer	Bleeding, discharge, ulcer, noticed larvae	NR
2[71]	81	F	2005	China	Hong Kong	The elderly home	Mouth	NR	Bedridden, ulcer	Bleeding, noticed larvae	Death (septicemia)**
3[72]	79	F	2005	China	Hong Kong	The elderly home	Mouth	NR	Bedridden	Bleeding, chills, noticed larvae	Death (NR)**
4[73]	78	F	2005	China	Hong Kong	An extended care hospital	Mouth	Surgical removal	NR	Swollen, bleeding	Stable
5[74]	50	F	2005	China	Hong Kong	NR	Limb: left lower limb	NR	Ulcer	NR	Stable
6[75]	66	M	2006	China	Hong Kong	NR	Scalp	NR	Scalp wound	Noticed larvae	Stable
7[76]	49	M	2006	China	Hong Kong	NR	Perineal & inguinal regions: groin	NR	NR	Pain, noticed larvae	Stable
8[77]	79	M	2006	China	Hong Kong	The elderly home	Mouth	Surgical removal	Bedridden	Bleeding, ulcer, noticed larvae	Stable
9[78]	93	F	2007	China	Hong Kong	The elderly home	Mouth: right lower gum	Surgical removal	Bedridden, stroke	Bleeding, fever	Stable
10[79]	49	M	2010	China	Hong Kong	NR	Torso: right shoulder	NR	Nasopharyngeal carcinoma, slipped and fell into a valley, a shoulder wound	Noticed larvae	Stable
11[80]	31	F	2010	China	Hong Kong (Indonesian)	NR	Torso: back	NR	Infection: ruptured back mass, wound	Bleeding, discharge, noticed larvae	Stable
12[80]	93	F	2010	China	Hong Kong	The elderly home	Mouth	NR	Multiple underlying illnesses, bedridden, dementia, tube feeding	Fever, ulcer	Death (underlying illnesses)**
13[29]	78	F	2010	China	Hong Kong	The elderly home	Mouth	NR	Bedridden	Bleeding, noticed larvae	Discharged
14[29]	81	M	2010	China	Hong Kong	The elderly home	Torso: sacral sore	NR	Bedridden, dementia, multiple bed sores, infections	Noticed larvae	Death (NR)**
15[29]	80	F	2010	China	Hong Kong	The elderly home	Tracheostomy	NR	Bedridden, tracheostomy wound	Bleeding, noticed larvae	Stable
16[81]	89	F	2010	China	Hong Kong	NR	Limb: left foot	NR	Slipped and an injury over her left foot, infection: later developed gangrene, wound	Necrosis	Stable
17[81]	88	F	2010	China	Hong Kong	The elderly home	Mouth	NR	Bedridden, tube feeding	Bleeding, noticed larvae	Stable
18[25]	89	F	2011	China	Hong Kong	Home	Limb: right foot	Surgical removal, below knee amputation due to extensive ulceration	Multiple underlying illnesses, hypertension, stroke, diabetes mellitus, diabetic ulcer	Noticed larvae, severe tissues and/or bone destruction	Stable
19[82]	83	F	2011	China	Hong Kong	The elderly home	Limb: right foot	Manual removal, surgical removal	Peripheral vascular disease with chronic ulcers on both legs	Noticed larvae	Stable
20[83]	82	M	2011	China	Hong Kong	home	Limb: right toe	Manual removal	Multiple underlying illnesses, dementia, hypertension, stroke, right toe ulcer	Noticed larvae, fever	Recovery/discharged
21[84]	80	F	2011	China	Hong Kong	NR	Mouth: gum	Manual removal	Multiple underlying illnesses, bedridden	Bleeding, noticed larvae	Recovery/discharged
22[84]	82	M	2011	China	Hong Kong	The elderly home	Mouth	Manual removal	Multiple underlying illnesses, bedridden, stroke, dementia, hypertension, chest infection	Fever, swollen, noticed larvae	Death (Pneumonia)**
23[85]	94	F	2011	China	Hong Kong	The elderly home	Mouth	Manual removal	Dementia,bedridden	Fever, noticed larvae	Stable
24[86]	94	F	2011	China	Hong Kong	The elderly home	Ear	Manual removal	Multiple underlying illnesses, ear infection: chronic otitis media, hypertension, stroke, dementia, bedridden	Discharge, noticed larvae	Stable
25[87]	79	F	2011	China	Hong Kong	The elderly home	Mouth	Manual removal	Multiple underlying illnesses, tube feeding, hypertension, stroke, pneumonia	Fever, noticed larvae	Death (Pneumonia)**
26[30]	47	M	2012	China	Hong Kong	Home	Tracheostomy	Manual removal	Nasopharyngeal carcinoma	Fever, discharge, noticed larvae	Stable
27[88]	51	M	2012	China	Hong Kong	The elderly home	Limb: right big toe	Manual removal, surgical removal, antibiotics	Ulcer	Swollen, bleeding, fever, noticed larvae	Recovery/discharged
28[88]	84	M	2012	China	Hong Kong	The elderly home	Mouth	Manual removal	Multiple underlying illnesses	Fever, noticed larvae	Stable
29[88]	88	M	2012	China	Hong Kong	The elderly home	Mouth: upper lip, hard palate	NR	Multiple underlying illnesses	Noticed larvae, necrosis, swollen	Stable
30[89]	83	M	2013	China	Hong Kong	The elderly home	Limb: left forearm	NR	Multiple underlying illnesses, bedridden, ulcer	Noticed larvae	NR
31[89]	73	F	2013	China	Hong Kong	The elderly home	Mouth: upper palate	Manual removal	Multiple underlying illnesses, bedridden, tube feeding	Noticed larvae	Recovery/discharged
32[90]	69	M	2013	China	Hong Kong	The elderly home	Mouth: the hard palate	Manual removal	Multiple underlying illnesses, wheelchair bound, pneumonia	Fever, swollen, bleeding, necrosis	Death (NR)**
33[91]	90	F	2013	China	Hong Kong	The elderly home	Mouth: gum, the upper palate	Manual removal	Multiple underlying illnesses, bedridden, tube feeding, pneumonia	Swollen, bleeding, ulcer, noticed larvae	Death (Pneumonia)**
34[92]	80	F	2013	China	Hong Kong	The elderly home	Mouth	Surgical removal	Multiple underlying illnesses, bedridden, tube feeding	Noticed larvae	Stable, discharged
35[93]	91	M	2015	China	Hong Kong	The elderly home	Mouth	NR	Multiple underlying illnesses, bedridden, tube feeding	Feeding problem, noticed larvae, ulcer	Death (underlying illnesses)**
36[94]	67	M	2015	China	Hong Kong	RCHE	Mouth: right upper lip, palate	Manual removal	Multiple underlying illnesses, bedridden	Necrosis, bleeding, noticed larvae	Stable
37[95]	74	M	2015	China	Hong Kong	RCHE	Mouth	Antibiotics	Multiple underlying illnesses, chest infection, bedridden	Bleeding, noticed larvae	Death (Pneumonia)**
38[96]	95	M	2015	China	Hong Kong	RCHE	Mouth	NR	Multiple underlying illnesses, tube feeding, bedridden	Ulcer, noticed larvae	Death (NR)**
39[97]	67	M	2015	China	Hong Kong	RCHE	Mouth: right upper lip, upper palate	Manual removal	Multiple underlying illnesses, bedridden	Necrosis, bleeding, noticed larvae, ulcer	Stable
40[98]	71	M	2015	China	Hong Kong	RCHE	Mouth	NR	Multiple underlying illnesses, bedridden, chest infection	Bleeding, noticed larvae	Death (Pneumonia)**
41[99]	89	F	2016	China	Hong Kong	RCHE	Mouth	Manual removal	Multiple underlying illnesses, bedridden, tube feeding	Swollen, noticed larvae	Recovery/discharged

42[99]	88	M	2016	China	Hong Kong	RCHE	Mouth	NR	Multiple underlying illnesses, bedridden, tube feeding	Noticed larvae	Stable
43[100]	80	M	2016	China	Hong Kong	NR	Limb: left ankle	Manual removal	Multiple underlying illness, chronic left ankle ulcer	Noticed larvae	Stable
44[100]	95	F	2016	China	Hong Kong	RCHE	Limb: right big toe	NR	Multiple underlying illness, trauma	Noticed larvae	Recovery/discharged
45[101]	46	F	2016	China	Hong Kong	Home	Torso: right breast	Manual removal	Multiple underlying illnesses, right breast wound	Noticed larvae	Recovery/discharged
46[102]	88	M	2017	China	Hong Kong	RCHE	Mouth	Manual removal	Multiple underlying illnesses, debility	Noticed larvae	Stable
47[103]	65	M	2018	China	Hong Kong	Low, RCHE	Mouth: palate	Manual removal, antibiotics	Infection: left dental abscess, ulcer	Fever,swollen, extensive palatal ulcers, noticed larvae	Stable, discharged
48[103]	83	F	2018	China	Hong Kong	Low, RCHE	Mouth	Manual removal, antibiotics	Multiple underlying illnesses, large ulcer	Gum bleeding, a large ulcer, noticed larvae	Death (NR)**
49[53]	96	M	2005	China	Hong Kong	Nursing home	Mouth	Manual removal, antibiotics, suffocating agent: turpentine	Multiple underlying illnesses, carcinoma of the lung and prostate, dementia, bedridden, infection: severe pneumonia	Fever, noticed larvae, ulcer, discharge, necrosis	Death (Pneumonia)**
50[53]	85	M	2005	China	Hong Kong	Home	Face: tumour base	Manual removal, antibiotics, suffocating agent: turpentine	Multiple underlying illnesses, stroke, gout, hypertension, left facial basal cell carcinoma, ulcer, semi-bedridden	Ulcer, noticed larvae	Recovery/discharged
51[53]	90	F	2011	China	Hong Kong	Nursing home	Eye	Manual removal, antibiotics, suffocating agent: turpentine	Multiple underlying illnesses, Chronic obstructive pulmonary disease, stroke, dementia, bedridden, right eyelid squamous cell carcinoma, Infection: old tuberculosis and aspergillosis	Swollen, discharge, noticed larvae	Death (Myocardial infarction)**
52[48]	89	F	2002	China	Hong Kong	NR	Mouth	Manual removal, surgical removal, broad-spectrum antibiotics therapy	Multiple underlying illnesses, stroke, tube feeding, cariovascular disease, bedridden, Infection: pulmonary tuberculosis, herpes simplex stomatitis, chest infection	Fever, ulcer, noticed larvae	Death (Congestive heart failure and nosocomial chest infection)**
53[18]	90	F	2003	China	Hong Kong	RCHE	Eye: right orbit, right upper and lower eyelids	Surgical removal	Multiple underlying illnesses, COPD, dementia,bedridden, squamous cell carcinoma of eyelid with ulcer, infections: old tuberculosis and aspergillosis	Bleeding, discharge, noticed larvae, necrosis, severe tissues and/or bone destruction: eyeball and the ocular tissues destroyed	Death (COPD and Myocardial infarction)**
54[26]	89	F	2002	China	Hong Kong	Nursing home	Mouth: palate, jaw	Manual removal, surgical removal	Stroke, dementia, bedridden, tube feeding	Noticed larvae	Death (Pneumonia)**
55[26]	69	F	2002	China	Hong Kong	Nursing home	Mouth: palate, jaw	Manual removal, surgical removal	Stroke, dementia, bedridden, tube feeding, oral ulcer	Bleeding	Recovery/discharged
56[26]	88	M	2002	China	Hong Kong	Nursing home	Mouth: palate, jaw	Manual removal, surgical removal	Dementia, bedridden, tube feeding	Noticed larvae, ulcer, severe tissues and/or bone destruction: extensive destroy of tissues, bone and teeth	Recovery/discharged
57[26]	77	F	2002	China	Hong Kong	Nursing home	Mouth: palate, subclavian sinus	NR	Dementia, bedridden, tube feeding	Noticed larvae, bleeding	Death (Pneumonia)**
58[26]	79	F	2003	China	Hong Kong	Nursing home	Perineal & inguinal regions: labia majora	Manual removal	Hypertension, dementia, bedridden, tube feeding, wound: sacral bed sore	Noticed larvae	Recovery/discharged
59[26]	71	M	2003	China	Hong Kong	Nursing home	Limb: foot	Manual removal, antibiotics, surgical removal and toe amputation	Diabetes mellitus, diabetic foot ulcer, blindness	Fever, discharge, foul smell, noticed larvae, severe tissues and/or bone destruction	Recovery/discharged
60[26]	96	M	2003	China	Hong Kong	Nursing home	Mouth: hard palate, maxillary sinuses	Manual removal, antibiotics	Carcinoma of the prostate, dementia, bedridden	Fever, secondary infection	Death (Pneumonia)**
61[104]	90	F	2004	China	Hong Kong	The elderly home	Mouth	Manual removal, surgical removal, antibiotics	Multiple underlying illnesses, bedridden, tube feeding, diabetes mellitus, hypertension, dementia and haemorrhagic stroke with limb contractures, bacterial infection	Fever, swollen, bleeding, noticed larvae, necrosis	Recovery
62[105]	68	M	2005	China	Hong Kong	A street sleeper, poor, low	Limb: the right ankle	Suffocating agent: turpentine, surgical removal	Ulcer	Ulcer, bleeding, noticed larvae	Recovery/discharged
63[105]	90	F	2005	China	Hong Kong	NR	Mouth	Manual removal, surgical removal, antibiotics	Mental illness, tube feeding, bedridden	Ulcer, noticed larvae, necrosis	Recovery/discharged
64[106]	86	F	2014	China	Hong Kong	Home, rural	Limb: left foot	Manual removal, surgical removal, antibiotics	Left foot erythema, ulcer, infection	Noticed larvae	Recovery
65[106]	74	M	2014	China	Hong Kong	Rural	Limb: right foot	Manual removal, surgical removal, antibiotics	End-stage renal disease, right foot trauma, bacterial infections	Noticed larvae	Recovery
66[106]	43	M	2014	China	Hong Kong	Low	Limb	Manual removal, surgical removal	Multiple underlying illnesses, neurological disorder: cognitive deficits, severe axonal mixed poluneuropathy involving his lower limbs, thyroid disorder: thyrotoxic hypokalemic periodic paralysis, postradioactive iodine hypothyroidism, infection: hemolytic streptococcal infective endocarditis, wound	Noticed larvae, necrosis	Recovery/discharged
67[106]	54	M	2014	China	Hong Kong	Low	Limb: left lower shin	Manual removal, surgical removal, antibiotics,	Chronic dermatitis	Noticed larvae, necrosis	Recovery/discharged
68[37]	61	F	2004	China	Hong Kong	NR	Torso: right breast	Manual removal, surgical removal	Invasive lobular carcinoma	Unbearable pain, foul smell, necrosis, ulcer, discharge, bleeding, noticed larvae	Recovery/discharged
69-85[107]	-	-	2002-2008	China	Hong Kong	-	-	-	-	-	-
86[14]	27	F	1976	China	Guangxi	Low	Perineal & inguinal regions	NR	Postpartum lochia	Discharge, pruritis: pruritus vulvae, tunnels	Recovery/discharged
87[14]	2	M	1980	China	Guixian, Guangxi	Low, rural	Ear	NR	Ear infection	Discharge, noticed larvae	Recovery/discharged
88[14]	6	F	1980	China	Tianyang, Guangxi	Low, rural	Ear	NR	Ear infection	Noticed larvae	Recovery/discharged
89[14]	37	F	1979	China	Daxin, Guangxi	Low, rural	Perineal & inguinal regions	NR	Infection: vulvitis and vaginis	Tunnels, honeycomb-like both side of the labia majora, noticed larvae	Recovery/discharged
90[14]	49	F	1978	China	Guiping, Guangxi	Low, rural	Nose	NR	Infection: atrophic rhinitis	Bleeding: rhinorrhagia, cold, fever, headache, noticed larvae	Recovery/discharged
91[14]	41	F	1973	China	Yining, Guangxi	Low, rural	Perineal & inguinal regions: the right of anus	NR	Miscarriage	Tunnels	Recovery/discharged
92[14]	1	M	1966	China	Nanning, Guangxi	City	Ear: left ear canal	NR	Ear infection	Noticed larvae	Recovery/discharged
93[14]	8	M	1959	China	Pinxiang, Guangxi	City	Nose	NR	Infection: rhinitis	Fever, discharge, foul smell, tunnels	Recovery/discharged
94[62]	56	F	1958	China	Nanning, Guangxi	Low, rural	Nose	Manual removal	Infection: rhinitis	Fever, discharge, foul smell, headache, ulcer, noticed larvae	Recovery/discharged
95[2]	NR	NR	1970	China	Yunnan	NR	Nose	NR	NR	NR	Recovery/discharged
96[*]	84	F	2009	China	Lufeng, Guangdong	Low, rural	Limb	Manual removal, suffocating agent	Hypertension, stroke	Large ulcer, severe pain, discharge, inflammation, oedema, sensation of moving larvae, noticed larvae, pruritus	Recovery/discharged
97[63]	54	F	2007	China	Jiangxi	Low, rural	Mouth, Nose	Manual removal, antibiotics	No	Huge ulcer between the hard and soft palate, connected with nose, necrosis, intense pain, eating difficulty, bleeding	Recovery/discharged

98[61]	49	F	1981	China	Quanzhou, Fujian	Low, rural	Perineal & inguinal regions	Surgical removal	NR	Pruritus vulvae, pain, discharge, edema, tunnels, noticed larvae	Recovery/discharged
99[64]	56	M	2015	China	Wei County, Hebei	Low, rural	Mouth	Manual removal	No	Pain, edema, redness, necrosis, ulcer, noticed larvae	Recovery/discharged
100[52]	45	F	2006	India	New Delhi	Low	Perineal & inguinal regions	Manual removal, suffocating agent: turpentine	Carcinoma cervix, bladder incontinence, ulcer	Pruritus, pain, noticed larvae	Discharged
101[108]	22	M	2014	India	Delhi	Low	Mouth	Manual removal, suffocating agent, antibiotics, insecticides: ivermectin	Neurological disorder and mental illness: cerebral plasy, severe mental retardation, quadriplegia, and kyphoscoliosis, seizure disorder	Noticed larvae, ulcer, swollen	Recovery/discharged
102[109]	71	NR	1990	India	Delhi	Low	Eye	Manual removal	Infection: herpes zoster opththalmicus on the face	NR	NR
103[56]	65	M	2016	India	Chhattisgarh	Low	Right eye	Manual removal, surgical removal, suffocating agent: turpentine oil, antibiotics, insecticides: ivermectin	Debility, immunocompromised, an empty orbital socket postevisceration	Pain, redness, discharge, swollen, necrosis, ulcer	Recovery/discharged
104[38]	65	F	1994	India	Shertallai	Low	Limb: left foot	Antibiotics	Infection:filarial, lymphoedema with dermatosclerosis and papillomatosis, ulcer	Fever, chill, pain, discharge, noticed larvae	Recovery
105[38]	60	M	1994	India	Shertallai	Low	Limb: left leg	Manual removal, suffocating agent: turpentine, antibiotics	Infection:filarial, lymphoedema with ulcers in both legs	Fever, chill, discharge	Recovery
106[38]	33	M	1994	India	Pondicherry	Low	Limb: left ankle	Manual removal, antibiotics	Infection:filarial lymphoedema in the left leg, ulcer	Severe pain, reddish, noticed larvae	Recovery
107[110]	60	M	2011	India	Punjub	Low	Mouth	Manual removal	Squamous cell carcinoma in oral cavity, ulcer, perforation of cheek	Severe pain, foul smell, discharge, noticed larvae	Recovery
108[110]	32	F	2011	India	Punjab	Low	Face, mouth	Manual removal, surgical removal, suffocating agent: turpentine	Squamous cell carcinoma in oral cavity, ulcer, perforation of cheek	Swollen, noticed larvae	Recovery
109[27]	50	F	2005	India	Punjub	Low	Limb	NR	Diabetes mellitus, diabetic foot, trauma, numbness	Noticed larvae	NR
110[27]	30	M	2006	India	Patiala	Low	Limb	Surgical removal, left foot amputation	Trauma, diabetes mellitus, diabetic foot, ulcer	High fever, discharge, noticed larvae, severe tissues and/or bone destruction: a bone deep gangrenous wound, necrosis	NR
111[51]	12	M	2012	India	NR	Low	Mouth	Manual removal, suffocating agent: cotton bud impregnated with turpentine	Neurological disorder	Swollen, tunnels, noticed larvae, foul smell	Recovery
112[111]	22	M	2011	India	Telangana	Low	Mouth	Manual removal	Mental illness	Pain, irritation, distress, bleeding, inflammation, abscess, ulcer	Recovery/discharged
113[111]	90	F	2011	India	Telangana	Low	Nose	Manual removal, suffocating agent	Hypertension, diabetes mellitus	Pain, distress, edema	Recovery/discharged
114[112]	4	F	2014	India	Madhya Pradesh	Low	Perineal & inguinal regions: Prolapsed rectum	Manual removal, suffocating agent	NR	Noticed larvae, ulceration,fever, inflammation	Recovery/discharged
115[31]	73	M	2013	India	Chennai	NR	Tracheostomy	Manual removal	Diabetes mellitus, carcinoma supraglottis, tracheostomy	Bleeding, discharge, ulcer, noticed larvae, edema, foul smell	Recovery/discharged
116[113]	33	M	2010	India	West Bengal	Low	Perineal & inguinal regions	Manual removal, antibiotics	Dermatitis, giant perigenital lesion, ulcer, mental illness	Noticed larvae, discharge	NR
117[50]	42	F	2015	India	Assam	Low	Eye: right eye	Manual removal, surgical removal, suffocating agent: turpentine, antibiotics	Trauma	Swollen, itch, bleeding, foul smell, discharge, noticed larvae	Recovery/discharged
118[114]	12	F	2009	India	Madhya Pradesh	NR	Mouth: gum	Manual removal, surgical removal, antibiotics	Gingival lesion, ulcer	Pain, swollen, bleeding, noticed larvae	Recovery
119[115]	70	F	2014	India	NR	Low	Mouth: anterior maxilla	Manual removal, surgical removal, suffocating agent: turpentine, antibiotics	Malnutrition	Swollen, pain, necrosis, severe tissues and/or bone destruction	Recovery
120[57]	12	M	2010	India	NR	Low	Mouth	Manual removal, suffocating agent: turpentine, insecticides: ivermectin	Neurological disorder: seizures, unconscious state, malnutrition, debility, tube feeding, ulcer, mouth breathing, incompetent lips	Swollen, reddish, tunnels, noticed larvae	Recovery/discharged
121[116]	40	M	2013	India	NR	Low	Mouth	Manual removal, surgical removal, insecticides: ivermectin, antibiotics	NR	Pain, swollen, noticed larvae, inflammation, fever, bone loss	Recovery/discharged
122[117]	14	M	2010	India	NR	Low	Mouth	Manual removal, suffocating agent: turpentine, antibiotics	Mental illness, neurological disorder: seizure, ulcer	Swollen, severe pain, fever, apprehensive, necrosis, noticed larvae	Recovery/discharged
123[118]	51	F	2013	India	NR	Low	Mouth	Manual removal, surgical removal, suffocating agent: turpentine, antibiotics	Infection: filarial lymphedema and elephantiasis, leprosy, incompetent lips, advanced periodontal disease	Pain, sensation of moving larvae, noticed larvae, inflammation, discharge, tunnels, bone destruction in teeth	Recovery/discharged
124[54]	35	F	2012	India	NR	Low	Mouth	Manual removal, surgical removal, suffocating agent: cottons buds impregnated with turpentine oil, insecticides: ivermectin, antibiotics	Mental illness, mouth breathing	Severe pain, swollen, noticed larvae, foul smell, tunnels	Recovery/discharged
125[32]	46	F	2008	India	Ariankuppam	Low	Tracheostomy	Manual removal, suffocating agent: turpentine	Advanced carcinoma of hypopharynx and neck, tracheostomy	Noticed larvae	NR
126[32]	52	F	2008	India	Tamil Nadu	Low	Tracheostomy	Manual removal, surgical removal, antibiotics	Carcinoma of larynx, tracheostomy, bacterial infections	Noticed larvae	Discharged
127[32]	62	M	2008	India	Tamil Nadu	Low	Face	NR	Carcinoma of cheek	Discharge, foul smell	NR
128[32]	75	M	2008	India	Tamil Nadu	Low	Mouth	NR	Ulcer, lip cancer	Bleeding, discharge, noticed larvae	NR
129[16]	30	M	1991	India	NR	Low	Limb: lower left leg	Manual removal	Ulcer, trauma, drug addict (heroin), infection: active cellulitis	Mixed infestation by *Chrysomya bezziana* and *Sarcophaga sp.,* severe pain, noticed larvae, foul smell, discharge, severe tissues and/or bone destruction: a bone-beep non-healing ulcer with 10cm×10cm in size	NR
130[119]	14	M	2011	India	NR	Low	Perineal & inguinal regions	Manual removal, antibiotics, suffocating agent: paraffin	Infections: perianal codylomata acuminata, bacterial infections	Pain, discharge, itch, foul smell, bleeding, necrosis, noticed larvae	NR
131[120]	32	F	2011	India	NR	Low	Mouth: hard palate and gums	Manual removal, insecticides: ivermectin, surgical removal	Mental illness, mouth breathing	Extensive necrosis, bleeding, foul smell, pain, swollen	Recovery/discharged
132[121]	-	-	1971	India	-		Mouth	-	-	-	-
133[33]	78	M	2011	India	NR	Low	Tracheostomy	Manual removal, surgical removal, suffocating agent: turpentine soaked gauze (but need caution while using it around the tracheostoma, a high risk of chemical pneumonitis), antibiotics	Head injury, trauma, tracheostomy, respiratory distress, bacterial infections	Foul smell, bleeding, discharge, noticed larvae	Recovery/discharged
134[19]	80	F	1990	India	NR	Low	Eye	Manual removal,suffocating agent: turpentine, antibiotics	No	Bleeding, discharge, noticed larvae, inflammation, ulcer, foul smell, severe tissues and/or bone destruction: complete loss globe in two days	Recovery/discharged
135[46]	50	F	2018	India	NR	Rural, low	Nose	Manual removal, suffocating agent, antibiotic	Diabetes mellitus, nasal obstruction and epistaxis	Noticed larvae, bleeding, necrosis, discharge, tunnels, severe tissues and/or bone destruction: destroy of bony septum	Recovery/discharged
136[5]	35	F	1909	India	Assam	Low	Nose, eye, face, mouth	Thousands of screw-worms with several generations were found, removed by manual removal (including nose, eye, face and mouth), using suffocating agent(nose): turpentine, treatment: nasal douche	Multiple underlying illnesses, Infections: ankylostomiasis, malaria, bronchitis, anaemia, asthma	Noticed larvae, fever, acute inflammation, Tunnels,oedema, bleeding, foul smell, discharge, pain, necrosis, severe tissues and/or bone destruction: from below the nostrils to halfway up to forehead. eyelids and cheeks, including subcutaneous tissue, muscles, mucous membrane, cartilage, periosteum and even bone	Death (exhaustion with rapid and heavy tissue destruction caused by a large number of maggots, and other underlying

											diseases worsen)**
137[55]	8	M	2018	India	NR	NR	Mouth: the posterior region of the jaw	Surgical removal, manual removal, suffocating agent: turpentine soaked gauze, antibiotics: amoxycillin, insecticides: ivermectin	Mental illness: mental retardation, malnutrition, infection: chronic pericoronitis	Noticed larvae, necrosis	Recovery/discharged
138[122]	3	F	2019	India	Punjab	Low	Scalp	Manual removal, suffocating agent: turpentine liniment, antibiotics	Trauma, anemia, infection	High fever, severe pain, noticed larvae	Recovery/discharged
139[19]	80	F	1990	India	NR	Low	Eye: globe	Manual removal, surgical removal, suffocating agent: turpentine	Trauma, infection: endophthalmitis	A large ulcer of about 6 m× 2.5 cm, inflammation, bleeding, discharge, foul smell, Severe tissues and/or bone destruction: the globe completely destroyed in two days	Discharged
140[34]	3	F	2011	Iran	Hormozgan	Low	Pharyngostomy	Surgical removal, antibiotics	Ingested aluminum hydroxide powder by accident, severe burns of oral cavity and esophagus, pharyngostomy tube	Noticed larvae, swollen	Stable
141[20]	55	F	2013	Iran	Mazandaran	Low	Eye: left eye	Surgical removal	Basal cell carcinoma	Noticed larvae, pain, severe tissues and/or bone destruction: left eye completely destroyed	NR
142[123]	55	M	2002	Iran	Kashan	Low	Ear	Surgical removal, suffocating agent: chloroform-containing sterile oil, antibiotics	Prolonged ear infection	Pain, discharge	Recovery/discharged
143[21]	87	F	2010	Iran	Khuzestan	Low	Eye: left orbit	Manual removal, surgical removal, antibiotics	Multiple underlying illnesses, trauma, cancer, eyelid surgical removal due to skin cancer, bedridden, back injury, bladder and bowel incontinence	Severe pain and necrosis, noticed larvae, swollen, redness, severe tissues and/or bone destruction: eyelids and eyeball completely destroyed	Recovery/discharged
144[35]	87	M	2009	Iran	Fars	NR	Limb	NR	Multiple underlying illnesses, diabetes mellitus, diabetic feet ulcers, cardiovascular diseases	Severe itch	NR
145[35]	18	M	2009	Iran	Fars	Low	Mouth	Manual removal	Mental illness and neurological disorder: congenital cerebral palsy with quadriplegia and mental retardation, severe failure to thrive, oral lesion, ulcer	Pain, noticed larvae	Recovery/discharged
146[124]	5	M	2007	Iran	Hormozgan	Low	Scalp	Surgical removal	NR	Severe headache , agitation, severe tissue destruction, wound, noticed larvae	NR
147[47]	74	F	2015	Iran	Khorasan Razavi	NR	Nose	Surgical removal, antibiotics	COPD, infections: pneumonia, severe sepsis	Noticed larvae in both nostrils, bleeding, perforation, fever	Death (underlying diseases worsen, severe sepsis, septic shock, multiorgan failure)**
148[22]	90	F	2000	Iran	Andimeshk town	Low	Eye	Manual removal, surgical removal and orbital exenteration	Leukomatous lesion, the eyelid basal cell carcinoma, chronic eye infection	Severe tissue/bone destruction: ocluar rupture and larvae penetrate deep enough to involve the optic canal, noticed larvae, severe swollen, discharge, bleeding	NR
149[125]	36	F	1978	Iran	NR	NR	Perineal & inguinal regions	Manual removal	Infection: appendicitis	Severe abdominal pain, dysuria, noticed larvae	Recovery/discharged
150[126]	-	-	1993	Iran	-		-	-	-	-	-
151[127]	89	M	2012	Iran	NR	NR	Mouth	Manual removal, antibiotics	Multiple underlying illnesses, non-Hodgkin lymphoma, dementia, infection: sepsis, immunocompromised	Fever, necrosis, noticed larvae	Death (Sepsis)**
152[23]	65	F	2009	Iran	NR	Low	Face, nose, nosal sinuses, mouth: upper lip, eye: left orbit	Manual removal, antibiotics,	Facial squamous cell carcinoma, wound	Bleeding, pain, discharge, noticed larvae, ulcer, severe tissues and/or bone destruction: soft tissues malignancy, involving left orbit, nose, maxillary sinus and sphenoid bone	Discharged
153[28]	65	M	2002	Malaysia	Kuala Lumpur	Low, rural	Limb: right big toe	Manual removal, surgical removal and amputation of the big toe	Diabetic mellitus, diabetic foot ulcer	Inflammation, oedema, bleeding, foul smell, discharge, noticed larvae, severe tissues and/or bone destruction	Recovery/discharged
154[128]	80	F	2005	Malaysia	Perak	-	Nose	-	-	Bleeding: epistaxis, headache, noticed larvae	-
155[129]	76	F	1980	Malaysia	-	Low	Perineal & inguinal regions	-	Carcinoma of the rectum, ulcer	Foul smell	-
156[130]	41	M	2006	Malaysia	NR	NR	Ear	Manual removal, antibiotics: amoxicillin	Mental illness	Pain, bleeding, discharge, noticed larvae, sensation of moving larvae, inflammation	Recovery/discharged
157[15]	27	F	2012	Malaysia	Saunsar village	Low, rural	Perineal & inguinal regions: vagina	Manual removal, suffocating agent: a pack soaked with turpentine oil kept in vagina, antibiotics	HIV and hepatitis virus infections, immunocompromised state, wound: left mediolateral episiotomy wound postnatal period	Pain, irritation, edema, wound, noticed larvae	Recovery/discharged
158[17]	55	M	2012	Travel to Malaysia	Island offshore of Langkawi	middle class	Scalp	Manual removal, antibiotics	Chronic seborrhoeic eczema	Pain, bleeding, noticed larvae, ulceration, sensation of moving larvae, itch	Recovery/discharged
159[131]	46	F	1952	Sri Lanka	NR	Low	Nose	Manual removal, suffocating agent: oil, plugging the nose with dextrose solution	Infection: sinusitis and rhinitis atrophicans, anemia	Discharge, foul smell, headache, bleeding, fever, edema, severe tissue and/or bone destruction: septum defect, noticed larvae	Recovery/discharged
160[131]	30	F	1951	Sri Lanka	NR	NR	Ear	Surgical removal, Manual removal, wound dressed with plugs soaked in 10% dextrose solution (dress every day)	Infection: otitis media chronica, conductive and nerve deafness, cholesteatomatous cavity	Discharge, foul smell, bone destruction, pain	Recovery/discharged
161[36]	73	F	1997	Sri Lanka	Colombo	Low	Limb: fourth toe, right foot	Manual removal, suffocating agent: turpentine	Dermatitis, mental illness	NR	Discharged
162[36]	82	F	1997	Sri Lanka	Colombo	Low	Limb: right heel	Manual removal, suffocating agent: turpentine	Dermatitis	NR	Discharged
163[36]	64	M	1997	Sri Lanka	Colombo	Low	Limb: right big toe	Manual removal, suffocating agent: turpentine	Dermatitis	NR	Discharged
164[36]	11	F	1997	Sri Lanka	Colombo	Low	Scalp	Manual removal, suffocating agent: turpentine	Infection: scalp sepsis, ectoparasite: pediculosis, mental illness: mental retardation	NR	Discharged
165[36]	55	F	1997	Sri Lanka	Colombo	Low	Limb: dorsum of left foot	Manual removal, suffocating agent: turpentine	Dermatitis	NR	Discharged
166[36]	73	M	1997	Sri Lanka	Colombo	Low	Limb: right foot	Manual removal, suffocating agent: turpentine	Ulcer, infections: leprosy, dermatitis	NR	Discharged
167[36]	94	M	1997	Sri Lanka	Colombo	Low	Limb: left foot	Manual removal, suffocating agent: turpentine	Dermatitis, senile debility	NR	Discharged
168[36]	60	M	1997	Sri Lanka	Colombo	Low	Limb: foot	Manual removal, suffocating agent: turpentine	Ulcer	NR	Discharged
169[36]	49	M	1997	Sri Lanka	Colombo	low	Limb: foot	Manual removal, suffocating agent: turpentine	Diabetes mellitus, diabetic ulcer	NR	Discharged
170[36]	39	M	1997	Sri Lanka	Colombo	Low	Limb: foot	Manual removal, suffocating agent: turpentine	Trauma	NR	Discharged
171[36]	65	F	1997	Sri Lanka	Colombo	Low	Limb: foot	Manual removal, suffocating agent: turpentine	Mental illness	NR	Discharged
172[36]	42	M	1997	Sri Lanka	Colombo	Low	NR	Manual removal, suffocating agent: turpentine	NR	NR	Discharged
173[36]	73	M	1997	Sri Lanka	Colombo	Low	NR	Manual removal, suffocating agent: turpentine	NR	NR	Discharged
174[36]	-	-	1997	Sri Lanka	Colombo	Low	NR	Manual removal, suffocating agent: turpentine	NR	NR	Discharged
175[37]	76	M	2016-2017	Sri Lanka	Penideniya, Kandy, KA	Low,poor, rural area	Limb: right heel	Manual removal	Diabetes mellitus, diabetic ulcer	NR	Discharged

176[37]	30	M	2016-2017	Sri Lanka	Kandy city, KA	Middle class	Limb: right heel	Manual removal	Diabetes mellitus, diabetic ulcer	NR	Discharged
177[37]	65	F	2016-2017	Sri Lanka	Thalathuoya, KA	Low, poor, rural area	Limb: left leg 2nd digit	Manual removal	Wound	NR	Discharged
178[37]	92	M	2016-2017	Sri Lanka	Muruthalawa, KA	Low, poor, rural area	Limb: right ankle	Manual removal	Mental illness, wound	NR	Discharged
179[37]	39	M	2016-2017	Sri Lanka	Wuraganthota, KA	Low, poor, rural area	Limb: left calf	Manual removal	Infection: filarial lymphedema, wound	NR	Discharged
180[37]	65	M	2016-2017	Sri Lanka	Galaha,KA	Low, poor, rural area	Torso: left buttock	Manual removal	Mental illness, wound	NR	Discharged
181[37]	54	F	2016-2017	Sri Lanka	Meepalawa,KA	Low, poor, rural area	Limb: right heel	Manual removal	Diabetes mellitus, diabetic ulcer	NR	Discharged
182[37]	39	M	2016-2017	Sri Lanka	Hakgala,BA (Badulla)	Low, poor, rural area	Limb: right foot	Manual removal	Diabetes mellitus, diabetic ulcer	NR	Discharged
183[37]	70	F	2016-2017	Sri Lanka	Agarapathana, NU (Nuwara eliya)	Low, poor, rural area	Limb: left calf	Manual removal	Wound	NR	Discharged
184[37]	70	M	2016-2017	Sri Lanka	Molagoda, KE (Keyalle)	Low, poor, rural area	Limb: right heel	Manual removal	Diabetes mellitus, diabetic ulcer	NR	Discharged
185[37]	79	M	2016-2017	Sri Lanka	Heeloya,BA	Low, poor, rural area	Perineal & inguinal regions: urogenital tract	Manual removal	Mental illness	NR	Discharged
186[37]	75	F	2016-2017	Sri Lanka	Kandy city, KA	Middle class	Limb: left great toe	Manual removal	Diabetes mellitus, diabetic ulcer	NR	Discharged
187[37]	69	M	2016-2017	Sri Lanka	Poonagala, BA	Low, poor, rural area	Limb: right foot	Manual removal	Diabetes mellitus, diabetic ulcer	NR	Discharged
188[37]	52	M	2016-2017	Sri Lanka	Dewaragampola, KE	Low, poor, rural area	Limb: left ankle	Manual removal	Diabetes mellitus, diabetic ulcer	NR	Discharged
189[37]	64	M	2016-2017	Sri Lanka	Pallepola,KA	Low, poor, rural area	Limb: light ankle	Manual removal	Mental illness, wound	NR	Discharged
190[37]	55	M	2016-2017	Sri Lanka	Owatta,KE	Low, poor, rural area	Limb: left ankle	Manual removal	Diabetes mellitus, diabetic ulcer	NR	Discharged
191[37]	53	M	2016-2017	Sri Lanka	Nayawela,KE	Low, poor, rural area	Limb: right leg 2-3 digits	Manual removal	Diabetes mellitus, diabetic ulcer	NR	Discharged
192[37]	61	M	2016-2017	Sri Lanka	Mawanella town, KE	Middle class	Limb: left ankle	Manual removal	Diabetes mellitus, diabetic ulcer	NR	Discharged
193[37]	64	M	2016-2017	Sri Lanka	Owaththa, KE	Low, poor, rural area	Limb: right calf	Manual removal	Diabetes mellitus, diabetic ulcer	NR	Discharged
194[37]	57	F	2016-2017	Sri Lanka	Nayawela,KE	Low, poor, rural area	Torso: right buttock	Manual removal	Diabetes mellitus, diabetic ulcer	NR	Discharged
195[37]	45	F	2016-2017	Sri Lanka	Uthuwankanda, KE	Low, poor, rural area	Limb: right ankle	Manual removal	Diabetes mellitus, diabetic ulcer	NR	Discharged
196[37]	45	F	2016-2017	Sri Lanka	Aranayake, KE	Low, poor, rural area	Scalp	Manual removal	Mental illness, wound	NR	Discharged
197[37]	48	F	2016-2017	Sri Lanka	Medawala, KA	Low, poor, rural area	Limb: right calf	Manual removal	Infection: filarial lymphedema, wound	NR	Discharged
198[37]	51	M	2016-2017	Sri Lanka	Nawalapitiya, KA	Low, poor, rural area	Torso: umbilical	Manual removal	Diabetes mellitus	NR	Discharged
199[37]	64	M	2016-2017	Sri Lanka	Pujapitiya,KA	Low, poor, rural area	Limb: right ankle	Manual removal	Diabetes mellitus, wound	NR	Discharged
200[37]	72	F	2016-2017	Sri Lanka	Galagedara,KA	Low, poor, rural area	Limb: left ankle	Manual removal	Diabetes mellitus, diabetic ulcer	NR	Discharged
201[37]	51	M	2016-2017	Sri Lanka	Aranayake,KA	Low, poor, rural area	Limb: right great toe	Manual removal	Wound	NR	Discharged
202[132]	55	F	2019	Indonesia	NR	Low	Nose, eye	Surgical removal, manual removal, antibiotics	Diabetes mellitus, diabetic neuropathy, infection: pansinusitis and right pleuropneumonia	Necrosis, ulcer, edema, severe tissues and/or bone destruction: destroy of nasal septum and bone, abscess	Discharged
203[49]	9	M	2008	Indonesia	Kalimantan	NR	Eye, ear	Surgical removal	Ear infection	Bleeding, discharge, noticed larvae, inflammation, wound	Recovery/discharged
204[133]	72	M	2009	Laos	Luang Namtha	Low	Limb: left lower leg	Surgical removal, antibiotics	Infections: chromoblastomycosis, cellulitis, leprosy, ectoparasite: leech bites, lesions	Noticed larvae, discharge, swollen, pain, ulcer	Recovery/discharged
205[134]	5	F	2009	Pakistan	Peshawar	Poor, low	Perineal & inguinal regions: urogenital tract	NR	NR	Pain, viginal itch, dysuria, noticed larvae on the bed, discharge, ulcer	Recovery/discharged
206[135]	-	-	1959	DRC	-	--	-	-	-	-	-
207[135]	-	-	1959	DRC	-	-	-	-	-	-	-
208-215†	NR	NR	2007,2008	Yemen	NR	NR	NR	NR	NR	NR	NR
216†	New born‡	NR	1949	PNG	Western Highlands	NR	NR	NR	NR	NR	NR
217†	NR	F	1951	PNG	New Ireland	NR	Perineal & inguinal regions	NR	NR	NR	NR
218†	NR	M	1951	PNG	New Ireland	NR	Torso	NR	NR	NR	NR
219†	14	M	1961	PNG	Easternn Highlands	NR	Face	NR	NR	NR	NR
220†	NR	M	1963	PNG	Easternn Highlands	NR	Ear	NR	NR	NR	NR
221†	NR	F	1963	PNG	Easternn Highlands	NR	Ear	NR	NR	NR	NR
222†	NR	F	1979	PNG	Morobe	NR	Eye	NR	NR	NR	NR
223†	5	M	1982	PNG	Western Province	NR	NR	NR	NR	NR	NR
224†	10	M	1983	PNG	Western Province	NR	Scalp	NR	NR	NR	NR

225[136]	NR	NR	1982	Saudi Arabia	NR	NR	Nose	NR	NR	NR	NR
226[24]	65	M	1985	Saudi Arabia	NR	Low	Eye: globe	Surgical removal, antibiotics	Multiple underlying illnesses, stroke, trauma, ulcers, bacterial infections, mental illness and neurological disorder: right-sided hemiparasis and mental retardation, superficial corneal pannus, stellate conjunctival scarring, and mature cataract	Increasing pain, inflammation, discharge, swollen, noticed larvae, severe tissues and/or bone destruction: totally destroyed orbit	Discharged
227[137]	94	F	2014	Thailand	Chiang Mai	Low	Eye: right orbit	Surgical removal	Bedridden, right eye infection	Right eyelid swollen, itch, ulcer, noticed larvae, bleeding, discharge, necrosis	Discharged
228-286[138-139]	-	-	1920-1922	India	-	-	-	-	-	-	-
287[140]	-	-	1929	Cote d'lvoire	-	-	Face	-	-	-	-
288[141]	-	-	1932	Kenya	-	-	Limb: foot	-	-	-	-
289[142]	-	-	1944	Uganda	-	-	Eye: orbit	-	-	-	-
290[142]	-	-	1944	Uganda	-	-	Nose	-	-	-	-
291[142]	-	-	1944	Uganda	-	-	Limb: foot	-	-	-	-

COPD=Chronic obstructive pulmonary disease; DRC=Democratic Republic of the Congo; PNG=Pupua New Guinea; RCHE=a residential care home for the elderly

* Case recorded by Zhou et al in Guangdong Province, China

† Spradbery et al Per.comm.

‡ Seveal strikes on vavels of new born children

**reported cause of death

References

70. Center For Health Protection. A case of *Chrysomya bezziana* infestation in a breast mass. Communicable Disease Watch. 2005;2:2.

71. Center For Health Protection. A local case of *Chrysomya bezziana* infestation. Communicable Disease Watch. 2005;2:95.

72. Center For Health Protection. A case of *Chrysomya bezziana* infestation. Communicable Disease Watch. 2005;2:83.

73. Center for Health Protection. Public reminded to guard against *Chrysomya bezziana* infestation. Available from: https://www.chp.gov.hk/en/features/2178.html.

74. Center for Health Protection. A case of human *Chrysomya bezziana* infestation confirmed. Available from: https://www.chp.gov.hk/en/features/2506.html.

75. Center for Health Protection. A case of human myiasis under investigation. Available from: https://www.chp.gov.hk/en/features/6811.html.

76. Center for Health Protection. Case of human *Chrysomya bezziana* infestation under investigation. Available from: http://www.info.gov.hk/gia/general/200604/07/P200604070245.html.

77. Center For Health Protection. A case of *Chrysomya bezziana* infestation. Communicable Disease Watch. 2006;3:22-23.

78. Center For Health Protection. A case of human myiasis reported. Communicable Disease Watch. 2007;4:22.

79. Center For Health Protection. A confirmed case of human myiasis. Communicable Disease Watch. 2010;7:66.

80. Center For Health Protection. Two confirmed cases of human myiasis. Communicable Disease Watch. 2010;7:91.

81. Center For Health Protection. Two confirmed cases of human myiasis. Communicable Disease Watch. 2010;7:105.

82. Center For Health Protection. A confirmed case of human myiasis. Communicable Disease Watch. 2011;8:5.

83. Center For Health Protection. A case of human myiasis. Communicable Disease Watch. 2011;8:66.

84. Center For Health Protection. Two cases of human myiasis. Communicable Disease Watch. 2011;8:99.

85. Center For Health Protection. A case of human myiasis. Communicable Disease Watch. 2011;8:15.

86. Center For Health Protection. A case of human myiasis. Communicable Disease Watch. 2011;8:24.

87. Center For Health Protection. A confirmed local recurrent case of human myiasis. Communicable Disease Watch. 2012;9:1.

88. Center For Health Protection. Four sporadic cases of human myiasis. Communicable Disease Watch. 2012;9:90-91.

89. Center For Health Protection. Two cases of human myiasis. Communicable Disease Watch. 2013;10:104.

90. Center For Health Protection. A case of human myiasis. Communicable Disease Watch. 2013;10:7.

91. Center For Health Protection. A case of human myiasis.Communicable Disease Watch. 2013;10:92.

92. Center For Health Protection. A case of human myiasis. Communicable Disease Watch. 2013;10:88.

93. Center For Health Protection. A sporadic case of human myiasis. Communicable Disease Watch. 2015;12:9.

94. Center For Health Protection. A case of human myiasis.Communicable Disease Watch. 2015;12:67.

95. Center For Health Protection. A case of human myiasis.Communicable Disease Watch. 2015;12:75.

96. Center For Health Protection. A confirm local case of human myiasis.Communicable Disease Watch. 2015;12:32.

97. Center For Health Protection. A cofirmed local case of human myiasi.Communicable Disease Watch. 2015;12:41.

98. Center For Health Protection. A sporadic local case of human myiasis in a residential care home for the elderly (RCHE). Communicable Disease Watch. 2015;12:110.

99. Center For Health Protection. Two cases of human myiasis. Communicable Disease Watch. 2016;13:7.

100. Center For Health Protection. Two local cases of human myiasis. Communicable Disease Watch. 2016;13:50.

101. Center For Health Protection. A sporadic case of human myiasis. Communicable Disease Watch. 2016;13:100.

102. Center For Health Protection. A sporadic case of human myiasis. Communicable Disease Watch. 2017;14:18.

103. Center For Health Protection. Two local cases of human myiasis. Communicable Disease Watch. 2018;15(19):84.

104. Poon T. Oral myiasis in Hong Kong - a case report. HK Pract. 2006;28:388-393.

105. Chan T, Yan K, Yien L, Yuen W. Oral and cutaneous myiases caused by *Chrysomya bezziana*. Surgical Practice. 2010;9:28-30.

106. Lam KH, Lui TH. Myiasis of the foot and leg caused by *Chrysomya bezziana*. J Foot Ankle Surg. 2014;53(1):88-91. doi: 10.1053/j.jfas.2013.05.001

107. Kwong A, Yiu WK, Chow LW, Wong S. *Chrysomya bezziana*: a rare infestation of the breast. Breast J. 2007;13(3):297-301. doi: 10.1111/j.1524-4741.2007.00426.x

108. Zachariah JE, Sehgal K, Dixit UB, Bhatia R. Oral myiasis: a case report. Spec Care Dentist. 2014;34(1):51-53. doi: 10.1111/scd.12016

109. Verma L, Pakrasi S, Kumar A, Sachdev MS, Mandal AK. External ophthalmomyiasis associated with herpes zoster ophthalmicus. Can J Ophthalmol. 1990;25(1):42-43.

110. Amandeep S, Devinder S, Rajiv J. *Chrysomya bezziana* (Diptera: Calliphoridae) as a causative agent of myiasis in patients suffering from oral cancer. Journal of Entomological Research. 2011;35:187-189.

111. Avula JK, Avula H, Arora N, Manchukonda UK, Vivekavardhan RN. Orofacial myiasis of the gingiva and nasal cavity: a report of two cases and general review. J Periodontol. 2011;82(9):1383-1388. doi: 10.1902/jop.2011.100724

112. Rathi S, Pednekar K, Pathak A, Singh P. Screw-worm myiasis of prolapsed rectum. Indian Pediatr. 2014;51(1):53-54.

113. Ghosh SK, Bandyopadhyay D, Sarkar S. Myiasis in a large perigenital seborrheic keratosis. Indian J Dermatol. 2010;55(3):305-306. doi: 10.4103/0019-5154.70699

114. Maheshwari V, Naidu S. Oral Myiasis caused by *Chrysomya bezziana* A case report. Peoples Journal of Scientific Research. 2010;3(2):25-26.

115. Aggarwal A, Daniel MJ, Shetty RS, Kumar BN, Sumalatha CH, Srikanth E, et al. Oral Myiasis Caused by *Chrysomya bezziana* in Anterior Maxilla. Case Reports in Dentistry. 2014;2014:1-4. doi: 10.1155/2014/518427

116. Jimson S, Prakash CA, Balachandran C, Raman M. Oral myiasis: case report. Indian J Dent Res. 2013;24(6):750-752. doi: 10.4103/0970-9290.127626

117. Sankari LS, Ramakrishnan K. Oral myiasis caused by *Chrysomya bezziana*. J Oral Maxillofac Pathol. 2010;14(1):16-18. doi: 10.4103/0973-029X.64304

118. Candamourty R, Venkatachalam S, Yuvaraj V, Sujee C. Oral myiasis in an adult associated with filariasis and Hansen's disease. J Nat Sci Biol Med. 2013;4(1):259-262. doi: 10.4103/0976-9668.107322

119. Pandhi D, Singal A, Das S. Myiasis arising in condylomata acuminata: an unusual presentation. Int J Std Aids. 2011; 22(4):238–240. https://doi.org/10.1258/ijsa.2010.010357

120. Kumar GV, Sowmya G, Shivananda S. *Chrysomya bezziana* oral myiasis. J Glob Infect Dis. 2011;3(4):393-395. doi: 10.4103/0974-777X.91066

121. Anand S, Taneja S. Oral myiasis, a case report. Armed Forces Med J India. 1971;27:277-278.

122. Singh A, Singh D. Traumatic Myiasis of the Scalp in a 3-year-old Patient- A Case of Neglected Health Care. Malaysian Journal of Medicine & Health Sciences. 2019;15(1):89-91.

123. Talari S, Yeganeh-Moghadam A, Dehghani R. *Chrysomya bezziana* infestation. Arch Iran Med. 2002;5(1):56-58.

124. Ahmadi MS, Nasirian H, Gheshmi AMN, Ershadi MRY. Human Extensive Head Skin Myiasis. Iran J Public Health. 2009;38(1):134-138.

125. Jdalayer T, Maleki M, Monghtaderi M. Human urogenital myiasis caused by *Chrysomyia bezziana*. Iran J Public Health. 1978;7:116-117.

126. El-Azazy OM. Old World screw-worm fly (*Chrysomyia bezziana*) in the Gulf. Vet Rec. 1993;132(10):256.

127. Alizadeh AM, Zamani N. Myiasis in an 89-year-old man with non-hodgkin lymphoma. J Arthropod Borne Dis. 2014;8(1):117-118.

128. Lee HL, Krishnasamy M, Jeffery J. A case of human nasopharyngeal myiasis caused by *Chrysomya bezziana* Villeneuve, 1914 (Diptera: Calliphoridae) in Malaysia. Trop Biomed. 2005;22(1):87-88.

129. Ramalingam S, Nurulhuda A, Bee LH. Urogenital myiasis caused by *Chrysomya bezziana* (Diptera: Calliphoridae) in peninsular Malaysia. The Southeast Asian journal of tropical medicine and public health. 1980;11(3):405-407.

130. Rohela M, Jamaiah I, Amir L, Nissapatorn V. A case of auricular myiasis in Malaysia. Southeast Asian J Trop Med Public Health. 2006;37 Suppl 3:91-94.

131. Bayer HG. Myiasis maligna of nose and ears in Ceylon; recommendation of a new treatment. AMA Arch Otolaryngol. 1954;59(1):104-107.

132. Lubis RR, Albar MY, Darlan DM. Massive orbital myiasis arising from nasal myiasis in an Indonesian patient with diabetes. American journal of ophthalmology case reports. 2019;13:147-150. doi: 10.1016/j.ajoc.2019.01.006

133. Slesak G, Inthalad S, Strobel M, Marschal M, Hall MJ, Newton PN. Chromoblastomycosis after a leech bite complicated by myiasis: a case report. Bmc Infect Dis. 2011;11:14. doi: 10.1186/1471-2334-11-14

134. Zaidi F, Ali N, Khisroon M. Urinogenital Myiasis From Blow Fly in a Pakistani Child. J Coll Physicians Surg Pak. 2016;26(6 Suppl):S35-S36.

135. Fain A, Magis P, Verdin G, Donkers J, Gobbels P. [On 2 cases of human myiasis produced by *Chrysomyia bezziana* Villeneuve in the Belgian Congo]. Ann Soc Belg Med Trop (1920). 1959;39:763-765.

136. Ansari M, Oertley R. Nasal myiasis due to Bezzi's blowfly (screw-worm): case report. Saudi Med J. 1982;3:275-278.

137. Ausayakhun S, Limsopatham K, Sanit S, Sukontason K, Sukontason KL. Orbital ophthalmomyiasis caused by *Chrysomya bezziana* in Thailand. Trop Biomed. 2018;35(1):288-292.

138. Patton W. Note on the occurrence of Passeromyia heterochaeta Villeneuve in India. Ind J Med Res. 1920;8:30.

139. Patton W. Some notes on Indian Calliphorinae VII. Additional cases of myiasis caused by the larvae of *Chrysomyia bezziana* Vill., together with some notes on the Diptera which cause myiasis in man and animals. Ind J Med Res. 1922;9:654.

140. Bouffard G, Legac P. Myiase a *Chrysomyia bezzianum* observe chez un indigene de la Cote d'Ivoire. Bull Soc Path Exot. 1929;22:48.

141. Symes C, Roberts J. A list of the Muscidae and Oestridae causing myiasis in man and animals in Kenya recorded at the medical research laboratory, Nairobi. E Afr Med J. 1932;9:18.

142. Hopkins G. Notes on myiasis especially in Uganda. E Afr Med J. 1944;21:258.
