# Supplementary material for: Human Chrysomya bezziana myiasis: A systematic review
Source: PLoS Negl Trop Dis. 2019 Oct 16;13(10):e0007391. doi: 10.1371/journal.pntd.0007391 (PMC6821133; doi:10.1371/journal.pntd.0007391)
Supplement: S2 Table — (PDF) [file pntd.0007391.s006.pdf]

S2 Table. The patients with *Chrysomya bezziana* myiasis were grouped by age.

Age group	Cases number worldwide	Percentage (%)	Cases number in Hong Kong	Percentage (%)
age ≤ 14	21	3.44	0	0
age 15-64	73	27.84	11	12.94
age ≥ 65	96	34.02	57	67.59
No report	101	34.71	17	20.00
Total	291	100	85	100
