# Supplementary material for: Human Chrysomya bezziana myiasis: A systematic review
Source: PLoS Negl Trop Dis. 2019 Oct 16;13(10):e0007391. doi: 10.1371/journal.pntd.0007391 (PMC6821133; doi:10.1371/journal.pntd.0007391)
Supplement: S3 Table — (PDF) [file pntd.0007391.s007.pdf]

S3 Table. Summary of main therapies recorded in human cases with *Chrysomya bezziana* myiasis worldwide.

Methods for removal of larvae	Mouth	Limb	Perineal & inguinal regions	Ear	Eye	Nose	Tracheostomy/ Pharyngostomy	Face	Scalp	Torso
Manual removal (n = 127)	42	46	8	3	11	8	5	4	4	5
Surgical removal (n = 50)	18	12	1	3	10	2	3	1	1	2
Suffocating agent (n = 43)	11	13	4	2	6	4	2	3	2	0
Antibiotics (n = 47)	19	8	3	2	8	5	3	2	2	0
