# Supplementary material for: Human Chrysomya bezziana myiasis: A systematic review
Source: PLoS Negl Trop Dis. 2019 Oct 16;13(10):e0007391. doi: 10.1371/journal.pntd.0007391 (PMC6821133; doi:10.1371/journal.pntd.0007391)
Supplement: S4 Table — (PDF) [file pntd.0007391.s008.pdf]

S4 Table. Health outcomes of human cases due to *Chrysomya bezziana*.

**Worldwide**	**Age group**				**Total**
**Outcome**	age ≤ 14	age 15-64	age ≥ 65	No report	
Recovery/discharged/stable	15	64	67	2	148
Death	0	1	22	0	23
No report	6	8	7	99	120
Total	21	73	96	101	291

**Hong Kong**	**Age group**				**Total**
**Outcome**	age ≤ 14	age 15-64	age ≥ 65	No report	
Recovery/discharged/stable	0	10	36	0	46
Death	0	0	20	0	20
No report	0	1	1	17	19
Total	0	11	57	17	85
