# Supplementary material for: Human Chrysomya bezziana myiasis: A systematic review
Source: PLoS Negl Trop Dis. 2019 Oct 16;13(10):e0007391. doi: 10.1371/journal.pntd.0007391 (PMC6821133; doi:10.1371/journal.pntd.0007391)
Supplement: S5 Table — (PDF) [file pntd.0007391.s009.pdf]

S5 Table. Distribution of *Chrysomya bezziana* recorded in the world

Author	Year	Province/District	Countries	Continent	Note
Zhong YH[143]	1994	Guangdong/Zhaoqing	China	Asia	-
Fan ZD[2]	1997	South China, Xizang/Motuo	China	Asia	-
Yao QG, et al[144]	1999	Gansu/Lanzhou	China	Asia	-
Xu BH[145]	2001	Fujian/Shaowu, Fuqing, Quanzhou, Huian, Anxi, Dehua, Jinjiang, Xiamen	China	Asia	-
Zhang YQ, et al[146]	2001	Guangxi	China	Asia	-
Zhang SH, et al[147]	2002	Guangdong/Shenzhen	China	Asia	-
Zhu W, et al[148]	2003	Guangxi	China	Asia	-
Li Q[149]	2006	Yunnan, Xizang, Hunan, Fujian, Taiwan, Guangdong, Guangxi, Hainan	China	Asia	-
Shi J, et al[150]	2006	Hebei/Qinhuangdao	China	Asia	-
Fei XD[151]	2011	Hunan, Fujian, Taiwan, Guangdong, Hainan, Guangxi, Yunnan, Xizang	China	Asia	-
Deng XR, et al[152]	2004,2005	Fujian/Longyan	China	Asia	-
Liang G, et al[153]	2010	Guangdong/Nansha	China	Asia	-
Lin F, et al[154]	2010	Fujian/Fuzhou	China	Asia	-
Duan HS, et al[155]	2010	Hubei	China	Asia	-
Xue WQ[156]	2006	Qinghai, Xizang, Fujian, Hainan, Taiwan, Guangxi, Yunnan, Guangdong	China	Asia	-
Liu ZJ, et al[157]	2011	Gansu/Lanzhou, Shannxi/Baoji, Meixian, Qishan, Zhouzhi, Huxian, Xi’an, Lantian, Weinan, Huayin, Tongguan, Huaxian, Taibai	China	Asia	-
Wang Y, et al[158]	2012	Gansu/Lanzhou	China	Asia	-
Feng Y[159]	2015	Sichuan/Ya’an	China	Asia	-
Wardhana A, et al[160]	2012b	Hong Kong	China	Asia	-
Ng KH, et al[48]	2003	Hong Kong	China	Asia	-
Ready PD, et al[161]	2009	Hong Kong	China	Asia	-
Norris KR and Murray MD[162]	1964	Taiwan	China	Asia	Present
Kloft WJ et al[163]	1981	-	Bahrain	Asia	Present, few occurrences
Hall MJ, et al[164]	2001	-	Bahrain	Asia	Present, few occurrences
James MT[165]	1947	-	Bangladesh	Asia	Present
James MT[165]	1947	-	Cambodia	Asia	Present
Norris KR and Murray MD[162]	1964	Andhra Pradesh, Chhattisgarh, Dadra and Nagar Haveli, Karnataka, Kerala, Madhya Pradesh, Maharashtra, Odisha, Tamil Nadu, West Bengal	India	Asia	Present
James MT[165]	1947	-	India	Asia	Present
Wardhana AH, et al[160]	2012b	Irian Jaya, Java, Kalimantan, Nusa, Tenggara, Sulawesi, Sumatra	Indonesia	Asia	Present

Wardhana AH, et al[166]	2014	Irian Jaya, Java, Kalimantan, Nusa, Tenggara, Sulawesi, Sumatra	Indonesia	Asia	Present
Norris KR and Murray MD[162]	1964	Nusa, Tenggara, Sulawesi, Sumatra	Indonesia	Asia	Present
Wardhana AH, et al[160]	2012b	Boushehr and Hormozoan Provinces	Iran	Asia	Present
Hall MJ, et al[164]	2001	Boushehr and Hormozoan Provinces	Iran	Asia	Present
Hall MJ, et al[167]	2009	Boushehr and Hormozoan Provinces	Iran	Asia	Present
Wardhana AH, et al[160]	2012b	Basrah, Karbala and Diayala Provinces	Iraq	Asia	Present
Hall MJ, et al[164]	2001	Basrah, Karbala and Diayala Provinces	Iraq	Asia	Present
Hall MJ, et al[167]	2009	Basrah, Karbala and Diayala Provinces	Iraq	Asia	Present
Rajapaksa N and Spradbery JP[59]	1989	-	Kuwait	Asia	Present
Hall MJ, et al[164]	2001	-	Kuwait	Asia	Present
James MT[165]	1947	-	Laos	Asia	Present
Wardhana AH, et al[160]	2012b	-	Malaysia	Asia	Present
Norris KR and Murray MD[162]	1964	Peninsular Malaysia	Malaysia	Asia	Present
James MT[165]	1947	-	Myanmar	Asia	Present
Norris KR and Murray MD[162]	1964	-	Myanmar	Asia	Present
Hall MJ, et al[164]	2001	-	Myanmar	Asia	Present
Wardhana AH, et al[160]	2012b	Al-Batina, Al-Shargiah and Interior Districts	Oman	Asia	Present
Spradbery JP, et al[168]	1992	Al-Batina, Al-Shargiah and Interior Districts	Oman	Asia	Present
Hall MJ, et al[167]	2009	Al-Batina, Al-Shargiah and Interior Districts	Oman	Asia	Present
James MT[165]	1947	-	Pakistan	Asia	Present
Hall MJ, et al[164]	2001	-	Pakistan	Asia	Present
James MT[165]	1947	-	Philippines	Asia	Present
Rajapaksa N and Spradbery JP[59]	1989	-	Qatar	Asia	Present
Wardhana AH, et al[160]	2012b	Al-Khari, Al-Muzahimiyah, Al-Ehsaa	Saudi Arabia	Asia	Present
Ansari M and Oertley R[136]	1982	Al-Khari, Al-Muzahimiyah, Al-Ehsaa	Saudi Arabia	Asia	Present
Hall MJ, et al[167]	2009	Al-Khari, Al-Muzahimiyah, Al-Ehsaa	Saudi Arabia	Asia	Present
James MT[165]	1947	-	Sri Lanka	Asia	Present
James MT[165]	1947	-	Thailand	Asia	Present
Spradbery JP and Kirk J[40]	1992	-	United Arab Emirates	Asia	Present
Hall MJ, et al[164]	2001	-	United Arab Emirates	Asia	Present
James MT[165]	1947	-	Vietnam	Asia	Present

Wardhana AH, et al[160]	2012b	-	Yemen	Asia	Localised
Robinson AS, et al[169]	2009	-	Yemen	Asia	Localised
Hall MJ, et al[164]	2001	-	Cameroon	Africa	Present
Hall MJ, et al[164]	2001	-	Chad	Africa	Present
James MT[165]	1947	-	Congo	Africa	Present
Rovere J[3]	1910	-	Congo Democratic Republic	Africa	Present
Hall MJ, et al[170]	2014	-	Congo Democratic Republic	Africa	Present
James MT[165]	1947	-	Côte d'Ivoire	Africa	Present
James MT[165]	1947	-	Equatorial Guinea	Africa	Present
Hall MJ, et al[164]	2001	Two localities: Gondar, Yabello	Ethiopia	Africa	Present
Fetene T and Worku N[171]	2009	Woreta, northwestern Ethiopa	Ethiopia	Africa	-
James MT[165]	1947	-	Gambia	Africa	Present
James MT[165]	1947	-	Guinea	Africa	Present
Norris KR and Murray MD[162]	1964	-	Guinea-Bissau	Africa	Present
James MT[165]	1947	-	Kenya	Africa	Present
Norris KR and Murray MD[162]	1964	-	Senegal	Africa	Present
Wardhana Ah, et al[160]	2012b	East Cape Province	South Africa	Africa	Present
Baker JA, et al[172]	1968	East Cape Province	South Africa	Africa	Present
Hall MJ, et al[170]	2014	East Cape Province	South Africa	Africa	Present
Hall MJ, et al[170]	2014	-	Sudan	Africa	Present
James MT[165]	1947	-	Swaziland	Africa	Present
Norris KR and Murray MD[162]	1964	-	Tanzania	Africa	Present
Hall MJ, et al[170]	2014	-	Tanzania	Africa	Present
James MT[165]	1947	Zanzibar	Tanzania	Africa	Present
James MT[165]	1947	-	Uganda	Africa	Present
James MT[165]	1947	-	Zambia	Africa	Present
Wardhana AH, et al[160]	2012b	-	Zimbabwe	Africa	Present
Cuthbertson A[173]	1933	-	Zimbabwe	Africa	Present
Hall MJ, et al[164]	2001	-	Zimbabwe	Africa	Present
Rajapaksa N and Spradbery JP[59]	1988	Australian Northern Territory, Port Darwin	Australia	Oceania	Absent, intercepted only
Norris KR and Murray MD[162]	1964	-	Papua New Guinea	Oceania	Present

Hall MJ et al[164]	2001	-	Papua New Guinea	Oceania	Present
Spradbery JP and Tozer RS[174]	2013	-	Papua New Guinea	Oceania	Present

Reference

143. Zhong Y. The investigation of flies in Zhaoqing City. Journal of Medical Pest Control. 1994;10:1-4.
144. Yao Q, Ma Z, Zhou K, Chen G, Li G. Investigation on fly population distribution and seasonal changing at Lanzhou Zhongchuan Air Port. Port Health Control. 1999;4:26-29.
145. Xu B. The investigation on the medical insects in Fujian Province III (Diptera: Scarthophagidae, Anthomyiidae, Fannidae, Calliphoridae, Sarcophagidae). Journal of Medical Pest Control. 2001;17:295-302.
146. Zhang Y, Huang W. Corrections and additions to a list of parasites for livestock and poultry in Guangxi Zhuang Autonomous Region. Guangxi Nongye Shengwu Kexue. 2001;20:223-230.
147. Zhang S, Liang Z, Liang G, Jia F, Chen Z, Zhang X, et al. Contribution to the flies of Shenzhen. Journal of Medical Pest Control. 2002;18:347-349.
148. Zhu W, Zhao Y, Bian D, Xue W. Study of calyptratae from Guangxi region, China (diptera). Journal of Shenyang Normal University (Natural Science). 2003;21:52-55.
149. Li Q. Comparative study on arthropod communities of different ecosystems in Yuanmou Arid-hot Valley, Yunnan. Ph.D. Thesis, Beijing Forestry University. 2006.
150. Shi J, Zhao Y, Wang S, Zhang G, Chang M, Liu J, et al. Studies on the family Calliphoridae in Hebei Province (Diptera: Cyclorrhapha). J Med Pest Contrl. 2006; 22:832–835.
151. Fei X. A review of Calliphoridae from China and apply to Forensic Entomolgoy. M.Sc. Thesis, Shenyang Normal University. 2011.
152. Deng X, Wei W, Wu S, He C. Investigation on fly density in urban area in Longyan from 2004 to 2005. Preventive Medicine Tribune. 2007;13:987-989.
153. Liang G, Jia F, Yan Q, Ye T. Calyptratae flies of Nansha Port (Diptera: Calyptratae). Journal of Environmental Entomology. 2010;32:295-299.
154. Lin F, Zhang S, Xu B. Investigation on fly population distribution at Fuzhou Changle International Airport. Strait Journal of Preventive Medicine. 2010;16: 28-30.
155. Duan H, Yang Z, Xu G, Zhou S, Wen X, Yu C, et al. Study on delimitation of zoogeographical regions in Hubei. Chinese Journal of Hygienic Insecticides & Equipments. 2010;16: 450-454.
156. Xue W. Flies in the Qinghai-Tibetan Plateau (Insect: Diptera). Beijing: Science Press; 2006:211.
157. Liu Z. List of vector species in northwest China. Beijing: Military Medical Science Press; 2011:77.
158. Wang Y, Li G, Huo Z. Species and geographical distribution of flies in Lanzhou City. Chin J Hyg Insect & Equip. 2012;18:232-234.
159. Feng Y. Species record and narration of Calliphoridae in Sichuan Province, China (Calliphoridae: Diptera). Chin J Vector Biol & Control. 2015;26: 398-403.
160. Wardhana A, Muharsini S, Ready PD, Cameron M, Hall M. Geographical characteristics of *Chrysomya bezziana* based on external morphology study. JITV. 2012;17(1):36-48.
161. Ready PD, Testa JM, Wardhana AH, Al-Izzi M, Khalaj M, Hall MJ. Phylogeography and recent emergence of the Old World screwworm fly, *Chrysomya bezziana*, based on mitochondrial and nuclear gene sequences. Med Vet Entomol. 2009;23(S1):43-50. doi: 10.1111/j.1365-2915.2008.00771.x.
162. Norris KR, Murray MD. Notes on the screw-worm fly *Chrysomya bezziana* (Diptera: Calliphoridae) as a pest of cattle in Papua New Guinea. CSIRO Division of Entomology Technical Paper. Melbourne, Australia. 1964;6:26.
163. Kloft WJ, Noll GF, Kloft ES. Introduction of *Chrysomya bezziana* Villeneuve (Dipt., Calliphoridae) into new geographical regions by "transit infestation". (Durch "Transitbefall" bewirkte Einschleppung von *Chrysomya bezziana* Villeneuve (Dipt., Calliphoridae) in neue geographische Regionen.) Mitteilungen der Deutschen Gesellschaft fur Allgemeine und Angewandte Entomologie. 1981;3(1/3):151-154.
164. Hall MJ, Edge W, Testa JM, Adams ZJ, Ready PD. Old World screwworm fly, *Chrysomya bezziana*, occurs as two geographical races. Med Vet Entomol. 2001;15(4): 393-402.
165. James MT. The Flies that cause Myiasis in Man. Washington, D.C.:U.S. Dept. of Agriculture; 1947:175.
166. Wardhana AH, Cecchi G, Muharsini S, Cameron MM, Ready PD, Hall MJ. Environmental and phylogeographical determinants of the distribution of the Old World screwworm fly in Indonesia. Acta Tropica. 2014; 138(Suppl):S62-S68. doi: 10.1016/j.actatropica.2014.06.001.
167. Hall MJ, Wardhana AH, Shahhosseini G, Adams ZJ, Ready PD. Genetic diversity of populations of Old World screwworm fly, *Chrysomya bezziana*, causing traumatic myiasis of livestock in the Gulf region and implications for control by sterile insect technique. Med Vet Entomol. 2009;23(Suppl):51-58. doi:

10.1111/j.1365-2915.2008.00778.x.

168. Spradbery JP. Studies on the prepupal and puparial stages of the Old World screw-worm fly, *Chrysomya bezziana* Villeneuve (Diptera: Calliphoridae). CSIRO Division of Entomology Technical Report. 1992;49:24 .

169. Robinson AS, Vreysen MJ, Hendrichs J, Feldmann U. Enabling technologies to improve area-wide integrated pest management programmes for the control of screwworms. Med Vet Entomol. 2009;23(Suppl):1-7. doi: 10.1111/j.1365-2915.2008.00769.x.

170. Hall MJ, MacLeod N, Wardhana AH. Use of wing morphometrics to identify populations of the Old World screwworm fly, *Chrysomya bezziana* (Diptera: Calliphoridae): a preliminary study of the utility of museum specimens. Acta Tropica. 2014;138(Suppl):S49-S55. doi: 10.1016/j.actatropica.2014.03.023.

171. Fetene T, Worku N. Public health importance of non-biting cyclorrhaphan files. Trans R Soc Trop. 2009;103(2):187-191. doi: 10.1016/j.trstmh.2008.08.010.

172. Baker JA, Mchardy WM, Thorburn JA, Thompson GE. *Chrysomya bezziana* Villeneuve-some observations on its occurrence and activity in the Eastern Cape Province. South African Veterinary Medical Association. 1968;39:3-11.

173. Cuthbertson A. The Habits and Life Histories of some Díptera in Southern Rhodesia. Proceedings and Transactions of the Rhodesia Scientific Association. 1933;32:81-111.

174. Spradbery P, Tozer RS. Trapping Old World screw-worm fly, *Chrysomya bezziana* Villeneuve (Diptera: Calliphoridae), in Papua New Guinea including the coastal border with Torres Strait. Australian Journal of Entomology. 2013;52(2):164-167. doi: 10.1111/aen.12007.
