# Supplementary material for: Human Chrysomya bezziana myiasis: A systematic review
Source: PLoS Negl Trop Dis. 2019 Oct 16;13(10):e0007391. doi: 10.1371/journal.pntd.0007391 (PMC6821133; doi:10.1371/journal.pntd.0007391)
Supplement: S6 Table — (PDF) [file pntd.0007391.s010.pdf]

S6 Table. A gender analysis in patients with *Chrysomya bezziana* myiasis

**Worldwide**	**age ≤ 14**	**age 15-64**	**age ≥ 65**	**No report**	**Total**	**Percentage (%)**
Female	7	35	51	3	96	32.99
Male	13	38	44	2	97	33.33
No report	1	0	1	96	98	33.68
Total	21	73	96	101	291	100.00

**Hong Kong**	**age ≤ 14**	**age 15-64**	**age ≥ 65**	**No report**	**Total**	**Percentage (%)**
Female	0	5	31	0	36	42.35
Male	0	6	26	0	32	37.65
No report	0	0	0	17	17	20.00
Total	0	11	57	17	85	100.00
