# Supplementary material for: Human Chrysomya bezziana myiasis: A systematic review
Source: PLoS Negl Trop Dis. 2019 Oct 16;13(10):e0007391. doi: 10.1371/journal.pntd.0007391 (PMC6821133; doi:10.1371/journal.pntd.0007391)
Supplement: S7 Table — (PDF) [file pntd.0007391.s011.pdf]

S7 Table. Analysis of the socioeconomic status of patients with *Chrysomya bezziana* myiasis

Low socioeconomic status	Cases number worldwide	Percentage (%)	Cases number in Hong Kong	Percentage (%)
Yes	154	52.92	52	61.18
No	11	3.78	5	5.89
No report	126	43.30	28	32.94
Total	291	100.00	85	100.00
