# Supplementary material for: Human Chrysomya bezziana myiasis: A systematic review
Source: PLoS Negl Trop Dis. 2019 Oct 16;13(10):e0007391. doi: 10.1371/journal.pntd.0007391 (PMC6821133; doi:10.1371/journal.pntd.0007391)
Supplement: S1 Appendix — (PDF) [file pntd.0007391.s012.pdf]

Appendix S1. Animal cases or outbreaks due to *Chrysomya bezziana* myiasis.

Animal myiasis worldwide

Since 1909 [3], animal cases and outbreaks due to *C. bezziana* have been recorded in 24 countries worldwide, 16 of them from Asia, including China, Saudi Arabia, Iran, India, Iraq, Oman, Yemen, Pakistan, Kuwait, Singapore, Sri Lanka, Myanmar, Indonesia, United Arab Emirates (UAE), Bahrain and Malaysia (S8 Table; Fig 6A), 7 from Africa, including Zimbabwe, Cameroon, Chad, Kenya, South Africa, Ethiopia, and Democratic Republic of Congo (DRC) [1], and the last from PNG, Oceania, which was sourced from investigations by Spradbery et al (2019) [175]. At least 19 species of domestic and wild animals have been reported to be affected by *C. bezziana* including cattle, sheep, goats, dogs, cats, horses, camels, buffaloes, donkeys, pigs, Sambar and Rusa deer, water buffalo, birds, wallabies, cus cus, rabbits, tree kangaroos, elephants, and chickens (S8 Table). Exotic animals in zoos in *C. bezziana*-endemic areas are also susceptible such as honey and polar bears, gnu, hyaena, lion, puma, rhinocerus and tapir at a zoo in Malaysia [176]. These animals, especially the domesticated species, may represent a high risk in transmitting the disease to humans.

In China, outbreaks of the disease among animals have been recorded in 8 provinces/province-level regions including Hainan, Fujian, Guangxi, Yunnan, Guizhou, and Gansu, as well as Hong Kong and Taiwan (Fig 6B; S8 Table). From 1987 to 1991, 513 animal cases occurred in Guizhou. In Fujian, there were 20 and 85 cattle cases, and 3 pig cases reported in 2001, 2007 and 2011, respectively. Meanwhile, in current decade animal myiasis outbreaks due to *C. bezziana* have been reported in Indonesia in 2006-2009, 2009-2012 and 2014, Yemen in 2008-2009, Pakistan in 2012-2015, Iraq in 2013-2014, Sri Lanka in 2014, India in 2014 and 2018, Saudi Arabia in 2015-2016, Malaysia and Singapore in 2017 (S8 Table).

S8 Table. Characteristics of animal cases or outbreaks due to *Chrysomya bezziana* myiasis recorded worldwide

Author	Year	Country	Province/District	Study design	Animal	Number animals	Note
Yan R, et al[177]	1952, 1964, 1981	China	Fujian	Record	Goat	-	-
Yan R, et al[177]	1952, 1964, 1981	China	Fujian	Record	Cattle	-	-
Yan R, et al[177]	1952, 1964, 1981	China	Fujian	Record	Pig	-	-
Yan R, et al[177]	1983	China	Fujian	Animal experiments	Rabbit	-	-
Yan R, et al[177]	1983	China	Fujian	Animal experiments	Guinea pig	-	-
Zhang H[178]	2007	China	Fujian	Case-series	Cattle	85	-
Lan Y, et al[179]	2001	China	Fujian	Case-series	Cattle	20	-
Lan Y, et al[179]	2001	China	Fujian	Case-series	Water buffalo	1	-
Liao W[180]	2011	China	Fujian	Case-series	Pigs	3	-

Wang X, et al[181]	1987-1991	China	Guizhou	Case-series	Cattle, goats and sheep	513	-
Wang X, et al[181]	1988	China	Guizhou	Record	Goat	-	-
Wei C[182]	1992	China	Guizhou	Record	Goat	-	-
Wei C[182]	1992	China	Guizhou	Record	Cattle	-	-
Ye Y[14]	1963	China	Guangxi	Case report	Cattle	-	-
Ye Y[14]	1965	China	Guangxi	Case report	Cattle	-	-
Department of Veterinary Medicine, Guangxi Agricultural College[183]	1978	China	Guangxi	Field investigation	Cattle	-	-
Gan Y[184]	Before 1980	China	Hainan	Record	Cattle	-	-
Gan Y[184]	Before 1980	China	Hainan	Record	Pig	-	-
Gan Y[184]	Before 1980	China	Taiwan	Record	Sheep	-	-
Fan Z[2]	1927,1943, and before 1997	China	Taiwan	Record	Cattle	-	-
Fan Z[2]	1927,1943, and before 1997	China	Taiwan	Record	Water buffalo	-	-
Fan Z[2]	1927,1943, and before 1997	China	Taiwan	Record	Pig	-	-
Fan Z[2]	Before 1997	China	Yunnan	Record	Pig	-	-
Fan Z[2]	Before 1997	China	Gansu	Record	Goat	-	-
Fan Z[2]	Before 1997	China	Gansu	Record	Pig	-	-
Chemonges-Nielsen S[185]	2003	China	Hong Kong	Case-series	Pet dogs	12	-
McNae J[186]	2001	China	Hong Kong	Cross-sectional	Dogs	59	-
Ready PD, et al[161]	2009	China	Hong Kong	Record	-	-	-
Alahmed AM[187]	2004	Saudi Arabia	Riyadh	Cross-sectional	Sheep	-	-
Abo-Shehada MN[188]	1999-2000	Saudi Arabia	Eastern Province	Cross-sectional	Sheep	151	-
Abo-Shehada MN[188]	1999-2000	Saudi Arabia	Eastern Province	Cross-sectional	Goats	154	-
Abo-Shehada MN[188]	1999-2000	Saudi Arabia	Eastern Province	Cross-sectional	Cattle	57	-

Abo-Shehada MN[188]	1999-2000	Saudi Arabia	Eastern Province	Cross-sectional	Camels	12	-
Abo-Shehada MN[188]	1999-2000	Saudi Arabia	Eastern Province	Cross-sectional	Horses	16	-
Abo-Shehada MN[188]	1999-2000	Saudi Arabia	Eastern Province	Cross-sectional	Donkeys	1	-
Abo-Shehada MN[188]	1999-2000	Saudi Arabia	Riyadh	Cross-sectional	Sheep	11	-
Abo-Shehada MN[188]	1999-2000	Saudi Arabia	Riyadh	Cross-sectional	Goats	0	-
Abo-Shehada MN[188]	1999-2000	Saudi Arabia	Riyadh	Cross-sectional	Cattle	4	-
Abo-Shehada MN[188]	1999-2000	Saudi Arabia	Riyadh	Cross-sectional	Camels	0	-
Abo-Shehada MN[188]	1999-2000	Saudi Arabia	Riyadh	Cross-sectional	Horses	0	-
Abo-Shehada MN[188]	1999-2000	Saudi Arabia	Riyadh	Cross-sectional	Donkeys	0	-
Abo-Shehada MN[188]	1999-2000	Saudi Arabia	Qasseim	Cross-sectional	Sheep	22	-
Abo-Shehada MN[188]	1999-2000	Saudi Arabia	Qasseim	Cross-sectional	Goats	10	-
Abo-Shehada MN[188]	1999-2000	Saudi Arabia	Qasseim	Cross-sectional	Cattle	2	-
Abo-Shehada MN[188]	1999-2000	Saudi Arabia	Qasseim	Cross-sectional	Camels	0	-
Abo-Shehada MN[188]	1999-2000	Saudi Arabia	Qasseim	Cross-sectional	Horses	0	-
Abo-Shehada MN[188]	1999-2000	Saudi Arabia	Qasseim	Cross-sectional	Donkeys	0	-
Hall MJ, et al[167]	2003-2006	Saudi Arabia	-	Cross-sectional	Sheep	16	-
Hall MJ, et al[167]	2003-2006	Saudi Arabia	-	Cross-sectional	Goats	10	-
Hall MJ, et al[167]	2003-2006	Saudi Arabia	-	Cross-sectional	Cattle	2	-
Hall MJ, et al[167]	2003-2006	Saudi Arabia	-	Cross-sectional	Buffalo	0	-
Hall MJ, et al[167]	2003-2006	Saudi Arabia	-	Cross-sectional	Horse	0	-
Hall MJ, et al[167]	2003-2006	Saudi Arabia	-	Cross-sectional	Camel	1	-
Hall MJ, et al[167]	2003-2006	Saudi Arabia	-	Cross-sectional	Dog	0	-
Salama O, et al[189]	2015-2016	Saudi Arabia	Qussim region	Cross-sectional	Sheep	129	-
Salama O, et al[189]	2015-2016	Saudi Arabia	Qussim region	Cross-sectional	Goats	18	-

Ready PD, et al[161]	2009	Iran	-	Record	-	-	-
Hall MJ, et al[167]	2003-2006	Iran	-	Cross-sectional	Sheep	18	-
Hall MJ, et al[167]	2003-2006	Iran	-	Cross-sectional	Goats	20	-
Hall MJ, et al[167]	2003-2006	Iran	-	Cross-sectional	Cattle	4	-
Hall MJ, et al[167]	2003-2006	Iran	-	Cross-sectional	Buffalo	0	-
Hall MJ, et al[167]	2003-2006	Iran	-	Cross-sectional	Horse	0	-
Hall MJ, et al[167]	2003-2006	Iran	-	Cross-sectional	Camel	0	-
Hall MJ, et al[167]	2003-2006	Iran	-	Cross-sectional	Dog	0	-
Navidpour S, et al[190]	1995	Iran	Khoozestan	Cross-sectional	Sheep	408	-
Navidpour S, et al[190]	1995	Iran	Khoozestan	Cross-sectional	Dogs	8	-
Soundararajan C, et al[191]	2018	India	Tamil Nadu state	Case report	Elephant	-	-
Bora S, et al[192]	2018	India	Assam	Cross-sectional	Broilers	180	-
Ready PD, et al[161]	2009	Iraq	-	Record	-	-	-
Hall MJ, et al[167]	2003-2006	Iraq	-	Cross-sectional	Sheep	24	-
Hall MJ, et al[167]	2003-2006	Iraq	-	Cross-sectional	Goats	4	-
Hall MJ, et al[167]	2003-2006	Iraq	-	Cross-sectional	Cattle	11	-
Hall MJ, et al[167]	2003-2006	Iraq	-	Cross-sectional	Buffalo	1	-
Hall MJ, et al[167]	2003-2006	Iraq	-	Cross-sectional	Horse	1	-
Hall MJ, et al[167]	2003-2006	Iraq	-	Cross-sectional	Camel	0	-
Hall MJ, et al[167]	2003-2006	Iraq	-	Cross-sectional	Dogs	4	-
Sigit SH, et al[193]	first recognised in 1938, 1981	Indonesia	widespread in Sulawesi	-	-	-	-
Sigit SH, et al[193]	first recognised in 1938, 1981	Indonesia	widespread in Java	-	-	-	-
Sigit SH, et al[193]	first recognised in 1938, 1981	Indonesia	widespread in Sumba	-	-	-	-
Sigit SH, et al[194]	1978	Indonesia	-	-	-	-	-

Basset CR, et al[195]	1982	Indonesia	-	-	Cattle	-	Despite intensive husbandry, losses of calves were frequent and on one property of 5,200 cattle, nearly 3,000 screw-worm cases were recorded during a single year
Wardhana AH, et al[196]	2006-2009	Indonesia	Kediri	Cross-sectional	Cattle	-	-
Wardhana AH, et al[196]	2006-2009	Indonesia	Kediri	Cross-sectional	Goat	-	-
Wardhana AH, et al[196]	2006-2009	Indonesia	Nationwide	Survey	Cattle	167	-
Wardhana AH, et al[196]	2006-2009	Indonesia	Nationwide	Survey	Sheep	15	-
Wardhana AH, et al[196]	2006-2009	Indonesia	Nationwide	Survey	Goats	57	-
Wardhana AH, et al[196]	2006-2009	Indonesia	Nationwide	Survey	Buffalo	1	-
Wardhana AH, et al[196]	2006-2009	Indonesia	Nationwide	Survey	Horses	5	-
Wardhana AH, et al[196]	2006-2009	Indonesia	Nationwide	Survey	Dogs	7	-
Wardhana AH, et al[196]	2006-2009	Indonesia	Nationwide	Survey	Pig	1	-
Wardhana AH, et al[196]	2006-2009	Indonesia	Nationwide	Survey	NR	2	-
Wardhana AH, et al[166]	2014	Indonesia	-	Review from the literature and other sources	Livestock	-	-
Ready PD, et al[161]	2009-2012	Indonesia	Java, Sumatra	-	Goat	-	-
Ready PD, et al[161]	2009-2012	Indonesia	Java, Sumatra	-	Cow	-	-
Ready PD, et al[161]	2009-2012	Indonesia	Java, Sumatra	-	Calf	-	-
Hall MJ, et al[167]	2003-2006	Oman	-	Cross-sectional	Sheep	5	-
Hall MJ, et al[167]	2003-2006	Oman	-	Cross-sectional	Goats	35	-
Hall MJ, et al[167]	2003-2006	Oman	-	Cross-sectional	Cattle	0	-
Hall MJ, et al[167]	2003-2006	Oman	-	Cross-sectional	Buffalo	0	-
Hall MJ, et al[167]	2003-2006	Oman	-	Cross-sectional	Horse	0	-
Hall MJ, et al[167]	2003-2006	Oman	-	Cross-sectional	Camel	0	-
Hall MJ, et al[167]	2003-2006	Oman	-	Cross-sectional	Dog	0	-

Spradbery JP, et al[197]	1992	Oman	-	-	Goats and sheep	53(93%,57)	-
Ready PD, et al[161]	2009	Papua New Guinea		Record	-	-	-
Spradbery JP*	1970	Papua New Guinea	-	-	Cattle	-	-
Spradbery JP*	1975-1982	Papua New Guinea	-	-	Sheep	751	-
Spradbery JP*	1967-1981	Papua New Guinea	-	-	Rusa Deer	7	-
Spradbery JP*	1970-1978	Papua New Guinea	-	-	Pigs	7	-
Spradbery JP*	1973-1977	Papua New Guinea	-	-	Horses	3	-
Spradbery JP*	1964	Papua New Guinea	-	-	Donkeys	1	-
Spradbery JP*	1975, 1980	Papua New Guinea	-	-	Goats	2	-
Spradbery JP*	1972-1980	Papua New Guinea	-	-	Dogs	6	-
Spradbery JP*	1976	Papua New Guinea	-	-	Cat	1	-
Spradbery JP*	1976	Papua New Guinea	-	-	Birds	2	-
Spradbery JP*	1977	Papua New Guinea	-	-	Wallaby	1	-
Spradbery JP*	1973, 1979	Papua New Guinea	-	-	Cus Cus	2	-
Norris K, et al[162]	1964	Papua New Guinea		-	Cattle?	53(95%,56)	-
Spradbery JP*	2008	Yemen	-	Record	Cattle	-	-
Spradbery JP*	2008	Yemen	-	Record	Sheep	-	-
Spradbery JP*	2008	Yemen	-	Record	Goats	-	-
Spradbery JP*	2008	Yemen	-	Record	Camels	-	-
Spradbery JP*	2008	Yemen	-	Record	Deer or gazelle	-	-
Spradbery JP*	2009	Yemen	-	Record	Cattle	-	-
Spradbery JP*	2009	Yemen	-	Record	Sheep	-	-
Spradbery JP*	2009	Yemen	-	Record	Goats	-	-
Spradbery JP*	2008	Yemen	-	Outbreak report	Oats	-	-

Spradbery JP*	2008	Yemen	-	Outbreak report	Sheep	-	-
Spradbery JP*	2008	Yemen	-	Outbreak report	Cattle	-	-
Spradbery JP*	2008	Yemen	-	Outbreak report	Camels	-	-
Spradbery JP*	2008	Yemen	-	Outbreak report	Donkeys	-	-
Spradbery JP*	2008	Yemen	Mahwit	Outbreak report	Cattle	180	-
Spradbery JP*	2008	Yemen	Mahwit	Outbreak report	Sheep	848	-
Spradbery JP*	2008	Yemen	Mahwit	Outbreak report	Goats	998	-
Spradbery JP*	2008	Yemen	Mahwit	Outbreak report	Camels	351	-
Spradbery JP*	2008	Yemen	Mahwit	Outbreak report	Donkeys	295	-
Spradbery JP*	2008	Yemen	-	Record	-	1500	-
Spradbery JP, et al[40]	1992	Kuwait	-	-	-	-	-
Ready PD, et al[161]	2009	Kuwait	-	Record	-	-	-
Soundararajan C, et al[198]	2007	India	Tamil Nadu	Cross-sectional	Sheep	127	-
Katoch R, et al[199]	2014	India	Jammu	Case report	Buffalo	1	-
Kumar R, et al[200]	1984	India	-	-	Cattle	-	Food rot and foot and mouth disease(FMD) lead to a significant increase in the incidence of *C. bezziana myiasis*
Ready PD, et al[161]	2009	United Arab Emirates	-	Record	-	-	-
Spradbery JP, et al[40]	1992	United Arab Emirates	Fujairah	Cross-sectional	Cows, calves, and heifers	62	-
Spradbery JP, et al[40]	1992	United Arab Emirates	-	-	-	-	-
Rajapaksa N et al[59]	1977, 1985	Australia	-	-	Sheep	-	-
Zaidi F, et al[201]	2012-2015	Pakistan	North West	Cross-sectional	-	-	-
Han H, et al[202]	2017	Malaysia	-	Case-series	Dogs	3	-
Basset CR, et al[195]	1984	Malaysia	-	-	Cattle	38(84%,45)	-
Basset CR, et al[195]	1982	Malaysia	-	-	Cattle	2957	-

Fadzil M, et al[203]	1971	Malaysia	-	-	Cattle	-	-
Rajamanickam C, et al[204]	1986	Malaysia	-	-	Cattle	124(95%,130)	-
Ready PD, et al[161]	2009	Malaysia	-	Record	-	-	-
Norval RA[205]	1978	Zimbabwe	-	-	Cattle	Cattle losses exceeding 250,000 in 1977/78	Wide spread of tick-borne diseases and associated screw-worm infestations
Cuthbertson A[173]	1933	Zimbabwe	-	-	Cattle	-	-
Cuthbertson A[173]	1933	Zimbabwe	-	-	Horses	-	-
Cuthbertson A[173]	1933	Zimbabwe	-	-	Dogs	-	-
Ready PD, et al[161]	2009	Zimbabwe	-	Record	Cattle, horses, goats, sheep, pigs, dogs	-	Larvae collected from wounds of several host species in 2002
Spradbery JP*	2017	Singapore	Singapore Zoo	Outbreak report	Rusa unicolor or Cervus unicolor	-	-
Fain A, et al[206]	1959	DRC	Uele	-	African elephants	-	-
Rovere A, et al[3]	1910	DRC	-	-	-	-	-
Baker JA, et al[172]	1968	South Africa	The Eastern Cape Province		Cattle	A huge number	-
Ready PD, et al[161]	2009	Bahrain	-	Record	-	-	-
Spradbery JP, et al[40]	1992	Bahrain	-	-	-	-	-
Ready PD, et al[161]	2009	Cameroon	-	Record	-	-	-
Ready PD, et al[161]	2009	Chad	-	Record	-	-	-
Ready PD, et al[161]	2009	Myanmar	-	Record	-	-	-
Bandara W, et al[207]	2014	Sri Lanka	Peradeniya	Cross-sectional	Dogs	294	-
Bandara W, et al[207]	2014	Sri Lanka	Peradeniya	Cross-sectional	Cats	5	-
Obanda V,et al[208]	2012	Kenya	Kigio Wildlife Conservancy, Nairobi	-	Elands (Taurotragus oryx)	-	-
Meemark N, et al[209]	1988	Thailand	Villages in the north and east of Thailand	Cross-sectional	NR	-	-
Zumpt F[210]	1965	Ethiopia	-	-	Cattle	-	-

* Spradbery et al. Pers.comm.

Reference

175. Spradbery J, Sands D, Tozer R. Distribution and pest status of the Old World screw-worm fly, *Chrysomya bezziana*, in Papua New Guinea and the threat to Australian livestock and wildlife. Austral Entomol;2019.

176. Spradbery J, Vanniasingham J. Incidence of the screw-worm fly, *Chrysomya bezziana* at the Zoo Negara, Malaysia. Malaysian Veterinary Journal. 1980;7:28-32.

177. Yan R, Wong Y. Studies on *Chrysomya bezziana* Villeneuve and the myiasis it produced. Wuyi Science Journal. 1983;3: 157-163.

178. Zhang H. Cases report of farm cattle myiasis. Academic Annual Meeting of the Fujian Animal Husbandry and Veterinary. 2009.

179. Lan Y, Zhong T. Cases report of farm cattle myiasis. Fujian Journal of Animal Husbandry and Veterinary. 2001;23: 17.

180. Liao W. Cases report of pig myiasis. Jiangxi Journal of Animal Husbandry & Veterinary Medicine. 2015;4: 303-301.

181. Wang X, Wang Y, Xie J, Liang X, Mo F. Myiasis due to *Chrysomya bezziana*. Guizhou Animal Science and Veterinary Medicine. 1992;16: 3-6.

182. Wei C. Zoonotic parasitic diseases in Guizhou Province (II). Guizhou Animal Science and Veterinary Medicine. 1992;17: 28-32.

183. Department of Veterinary Medicine GAC. Survey of cattle and sheep parasite species in Guangxi Province. Guangxi Agricultural Sciences. 1978;4: 54-59.

184. Gan Y. On the larvae of the Chinese species of the subfamily *Chrysomyinae* (Dipt. Calliphoridae). Dongwuxue Yanjiu. 1980;1: 179-196.

185. Chemonges-Nielsen S. *Chrysomya bezziana* in pet dogs in Hong Kong: a potential threat to Australia. Australian Veterinary Journal. 2003;81: 202-205.

186. McNae JC, Lewis SJ. Retrospective study of Old World screwworm fly (*Chrysomya bezziana*) myiasis in 59 dogs in Hong Kong over a one year period. Australian Veterinary Journal. 2004;82: 211-214.

187. Alahmed AM. Myiasis in sheep farms in Riyadh Region, Saudi Arabia. Journal of the Egyptian Society of Parasitology. 2004;34: 153-160.

188. Abo-Shehada MN. Incidence of *Chrysomya bezziana* screw-worm myiasis in Saudi Arabia, 1999/2000. Vet Rec. 2005;156: 354-356. doi: 0.1136/vr.156.11.354.

189. Osman Salama, Omar Hussein. Clinical and epidemiological studies on screwworm infestation in Qassim region, Saudi Arabia. Tropical Biomedicine. 2017;34(3): 936-943.

190. Navidpour S, Hoghooghi-Rad N, Goodarzi H, Pooladgar AR. Outbreak of *Chrysomya bezziana* in Khoozestan province, Iran. The Veterinary record. 1996;139: 217.

191. Soundararajan C, Prabhu K, Nagarajan K, Divya T. Wound and gastric myiasis due to *Chrysomya bezziana* and *Cobbaldia elephantis* and its pathological lesions in wild elephants in the Nilgiris hills of Tamil Nadu. J Parasit Dis. 2018. doi: 10.1007/s12639-018-1068-x.

192. Bora S, Hussain L, Das M, Islam S, Bulbul H. Fly strike in broilers in Kamrup district of Assam. Journal of Entomology and Zoology Studies. 2018;6(2): 2439-2442.

193. Sigit SH, Partoutomo S. Myiasis in Indonesia. Bulletin Off Int Epiz. 1981;93: 173-178.

194. Sigit SH. Masalah myiasis padasapi di Sulawesi selatan. Media Veterinar. 1978;3: 1-12.

195. Basset CR, Kadir SB. The screw-worm fly (*Chrysomya bezziana*) - an obstacle to large-scale beef production in Malaysia. Animal Production Health Tropics. 1982: 133-135.

196. Wardhana AH, Abadi I, Cameron MM, Ready PD, Hall MJ. Epidemiology of traumatic myiasis due to *Chrysomya bezziana* in Indonesia. JITV. 2018;23(1): 45-60. doi: 10.14334/jitv.v23il.1617.

197. Spradbery JP, Khanfar KA, Harpham D. Myiasis in the sultanate of Oman. Vet Rec. 1992;131(4): 76-77. doi: 10.1136/vr.131.4.76.

198. Soundararajan C, Nagarajan K, Prakash MA. Occurrence of aural myiasis in madras red sheep and its management by herbal medicine (Tamarind Seed Coat Powder). Indian Veterinary Journal. 2017;94: 78-79.

199. Katoch R, Godara R, Yadav A, Sharma S, Ahmad I. Occurrence of *Chrysomya bezziana* in a buffalo in Jammu. Journal of Parasitic Diseases: Official Organ of the Indian Society for Parasitology. 2014;38: 420-422.

200. Kumar R, Ruprah NS. Incidence and etiology of cutaneous myiasis in Domestic animals at Hissar. Indian Veterinary Journal. 1984;61: 918-921.

201. Zaidi F, Fatima SH, Gul A. Dataset of traumatic myiasis observed for three dominant screw worm species in North West Pakistan with first report of *Wohlfahrtia magnifica* (Schiner). Data in Brief . 2016;8: 1333-1337.

202. Han HS, Sharma R, Jeffery J, Noli C. *Chrysomya bezziana* (Diptera: Calliphoridae) infestation: case report of three dogs in Malaysia treated with spinosad/milbemycin. Veterinary Dermatology. 2017;28: 239-262.

203. Fadzil M, Jaafa A, Soon CT. *Chrysomya bezziana* Villeneuve- Some Observations on its activity in the Tekam Research Stat ion, Jerantut, West Malaysia. Kajian Vet Malaysia-Singapore.

1971;3: 10-14.

204. Rajamanickam C, Soon CT, Paramasvaran S. The prevalence of myiasis of domestic animals in peninsular Malaysia. Kajian Vet J. 1986;18: 153-157.

205. Norval RA. The effects of partial breakdown of dipping in African areas in Rhodesia. Rhodesian Veterinary Journal. 1978;9: 9-16.

206. Fain A, Magis P, Verdin G, Donkers J, Gobbels P. Sur deux cas de myiases humaines produites par *Chrysomya bezziana* Villeneuve au Congo belge. Ann Soc Belge Med Trop. 1959;39: 763.

207. Bandara W, Karunaratne W, Fuward R, Dangolla A, Yasakeerthi A. Myiasis in dogs and cats treated in two veterinary clinics in peradeniya, Sri Lanka. Journal of Entomology and Zoology Studies. 2016;4(6): 211-215.

208. Obanda V, Ndambiri E, Kingori E, Gakuya F, Lwande O, Alasaad S. Traumatic myiasis in free-ranging eland, reported from Kenya. Parasit Vectors. 2013;6: 89. doi: 10.1186/1756-3305-6-89.

209. Meemark N. The Development and evaluation of a village-based parasite control program for swamp buffalo and cattle in Northeast Thailand. M.Phil. Thesis, Massey University;1988.

210. Zumpt F. Myiasis in Man and Animals in the Old World. Butterworths, London; 1965.
